# CD63^+^ Cancer‐Associated Fibroblasts Confer Tamoxifen Resistance to Breast Cancer Cells through Exosomal miR‐22

**DOI:** 10.1002/advs.202002518

**Published:** 2020-09-24

**Authors:** Yuan Gao, Xiaoju Li, Cheng Zeng, Chenlin Liu, Qiang Hao, Weina Li, Kuo Zhang, Wangqian Zhang, Shuning Wang, Huadong Zhao, Dong Fan, Meng Li, Yingqi Zhang, Wei Zhang, Cun Zhang

**Affiliations:** ^1^ The State Key Laboratory of Cancer Biology Biotechnology Center School of Pharmacy The Fourth Military Medical University Xi'an 710032 P. R. China; ^2^ Institute of Material Medical School of Pharmacy The Fourth Military Medical University Xi'an 710032 P. R. China; ^3^ Department of General Surgery Tangdu Hospital The Fourth Military Medical University Xi'an 710038 P. R. China

**Keywords:** breast cancer, cancer‐associated fibroblasts, exosomes, miR‐22, tamoxifen

## Abstract

Tamoxifen remains the most effective treatment for estrogen receptor α (ERα)‐positive breast cancer. However, many patients still develop resistance to tamoxifen in association with metastatic recurrence, which presents a tremendous clinical challenge. To better understand tamoxifen resistance from the perspective of the tumor microenvironment, the whole microenvironment landscape is charted by single‐cell RNA sequencing and a new cancer‐associated fibroblast (CAF) subset, CD63^+^ CAFs, is identified that promotes tamoxifen resistance in breast cancer. Furthermore, it is discovered that CD63^+^ CAFs secrete exosomes rich in miR‐22, which can bind its targets, ER*α* and PTEN, to confer tamoxifen resistance on breast cancer cells. Additionally, it is found that the packaging of miR‐22 into CD63^+^ CAF‐derived exosomes is mediated by SFRS1. Furthermore, CD63 induces STAT3 activation to maintain the phenotype and function of CD63^+^ CAFs. Most importantly, the pharmacological blockade of CD63^+^ CAFs with a CD63‐neutralizing antibody or cRGD‐miR‐22‐sponge nanoparticles enhances the therapeutic effect of tamoxifen in breast cancer. In summary, the study reveals a novel subset of CD63^+^ CAFs that induces tamoxifen resistance in breast cancer via exosomal miR‐22, suggesting that CD63^+^ CAFs may be a novel therapeutic target to enhance tamoxifen sensitivity.

## Graphical abstract

Tamoxifen resistance is a severe clinical challenge in breast cancer treatment. In the present study, single‐cell RNA sequencing reveals a new subset of cancer‐associated fibroblast (CD63^+^ CAF) in the tumor microenvironment, which downregulates ER*α* and PTEN expression in breast cancer cells through exosomal miR‐22 and thus induces tamoxifen resistance.

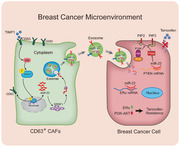

## 1 Introduction

Among all breast cancers, estrogen receptor *α* (ER*α*)‐positive tumors constitute the largest proportion (≈70%). The selective ER*α* modulator tamoxifen has been widely used as a first‐line adjuvant endocrine therapy for these tumors for decades and can significantly improve patient outcomes.^[^ ^1^ ^]^ Unfortunately, many breast cancer patients eventually develop tamoxifen resistance, which is associated with metastatic recurrence.^[^ ^2^ ^]^ Multiple mechanisms are responsible for tamoxifen resistance and the deregulation of ER*α* is the dominant one.^[^ ^3^ ^]^ Another important mechanism for tamoxifen resistance is the excessive activation of the PI3K‐Akt pathway.^[^ ^4^ ^]^ It is reported that PTEN is a major negative regulator of the PI3K‐Akt pathway by dephosphorylating PIP3 to PIP2 and MTDH can induce tamoxifen resistance through inhibiting PTEN expression and then activating the PI3K‐Akt pathway.^[^ ^5^, ^6^ ^]^ However, there are few clinically available strategies that can effectively reverse tamoxifen resistance. It is worth noting that previous studies on tamoxifen resistance have focused mainly on tumor cells without fully considering the tumor microenvironment (TME). Realistically, how tumor cells respond to therapy depends not solely on the genomic aberrations they harbor but also on the characteristics of the TME.^[^ ^7^ ^]^ Therefore, from a therapeutic perspective, it is urgent to elucidate the mechanism of tamoxifen resistance from the perspective of the whole TME.

Cancer‐associated fibroblasts (CAFs) constitute the major stromal components in many types of cancers, including breast cancer.^[^ ^8^, ^9^ ^]^ Accumulating evidence indicates that CAFs play key roles in promoting cancer progression,^[^ ^10^, ^11^ ^]^ which highlights the potential of CAFs as therapeutic targets. However, targeting the whole CAF population cannot effectively treat cancer and may even lead to cancer progression,^[^ ^12^, ^13^ ^]^ which suggests that CAFs represent a heterogeneous group of cells with diverse and opposing functions and that targeting all CAFs is not an appropriate treatment approach for new anticancer therapies. Therefore, developing better strategies to identify different CAF subpopulations and their corresponding functions is a critical unmet need for precision treatment.

Here, we have shown that there exists a specific CAF subset in the breast cancer microenvironment: CD63^+^ CAFs. Furthermore, we determined that CD63^+^ CAFs could promote tamoxifen resistance through exosomal miR‐22, which downregulated ER*α* and PTEN expression in breast cancer cells. Notably, we found that specifically inhibiting the function of CD63^+^ CAFs successfully enhanced the sensitivity of breast cancer to tamoxifen in an in vivo tumor model.

## 2 Results

### 2.1 Loss of Epithelial ER*α* Expression during Cancer Progression is Associated with a Poor Tamoxifen Response in Breast Cancer

**Figure 1:**
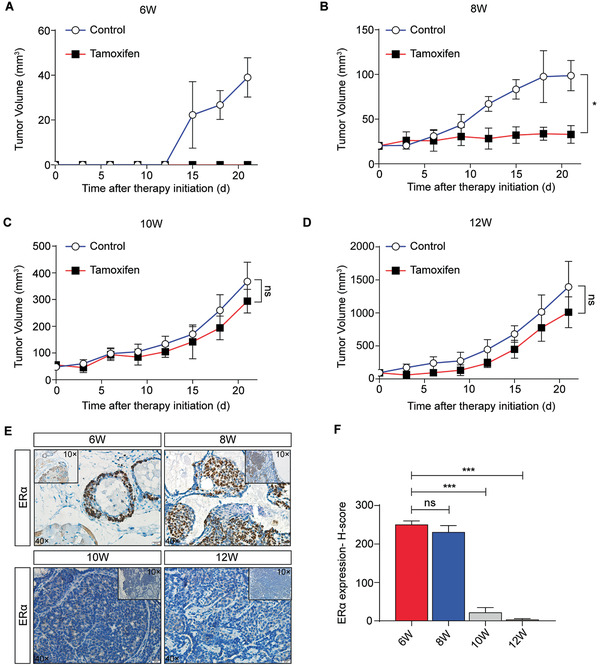
Loss of epithelial ER*α* expression during cancer progression is associated with poor tamoxifen response in breast cancer. A) Average volume of tumors from W6 MMTV‐PyMT mice. Mice were treated with oil vehicle or tamoxifen (*n* = 5). B) Average volume of tumors from W8 MMTV‐PyMT mice. Mice were treated with oil vehicle or tamoxifen (*n* = 5). C) Average volume of tumors from W10 MMTV‐PyMT mice. Mice were treated with oil vehicle or tamoxifen (*n* = 5). D) Average volume of tumors from W12 MMTV‐PyMT mice. Mice were treated with oil vehicle or tamoxifen (*n* = 5). E) Representative immunohistochemical staining for ER*α* in primary tumors of W6‐W12 MMTV‐PyMT mice. Scale bars = 100 µm (×10) and 20 µm (×40). F) The ER*α* expression score was quantified and analyzed in primary tumors from W6‐W12 MMTV‐PyMT mice. A–D,F) The data are shown as the means ± S.E.M. ns *p* > 0.05, ^:^ *p* < 0.05, ^:::^ *p* < 0.001. A–D) Unpaired *t*‐test. F) ANOVA with Dunnett's *t*‐test.

We used the transgenic polyoma middle T oncogene (PyMT)‐induced mouse model (FVB/N genetic background), which accurately reproduced the stepwise progression of human breast cancer.^[^ ^14^, ^15^ ^]^ We chose 6‐week‐old (W6), 8‐week‐old (W8), 10‐week‐old (W10), and 12‐week‐old (W12) MMTV‐PyMT mice to represent different breast histologies (hyperplasia, ductal carcinoma in situ, early invasive breast carcinoma and late invasive breast carcinoma, respectively) in breast cancer development^[^ ^16^ ^]^ and treated them with tamoxifen. The results showed that primary tumors from W6 and W8 mice were sensitive to tamoxifen (**Figure** **1**A,B), whereas primary tumors from W10 and W12 mice were not (Figure 1C,D). ER*α* expression has been reported to be a crucial determinant of the response to tamoxifen therapy;^[^ ^17^, ^18^ ^]^ therefore, we detected ER*α* expression in the primary tumors of W6, W8, W10, and W12 MMTV‐PyMT mice. ER*α* expression was high in the primary tumors of W6 and W8 mice, but a loss of epithelial ER*α* expression was observed in the primary tumors of W10 and W12 MMTV‐PyMT mice (Figure 1E,F). These results indicate that certain factors in the TME may induce tamoxifen resistance by regulating ER*α* expression during breast cancer progression.

### 2.2 Single‐Cell Sequencing Reveals that CAFs are Associated with Poor Tamoxifen Response in Breast Cancer

**Figure 2:**
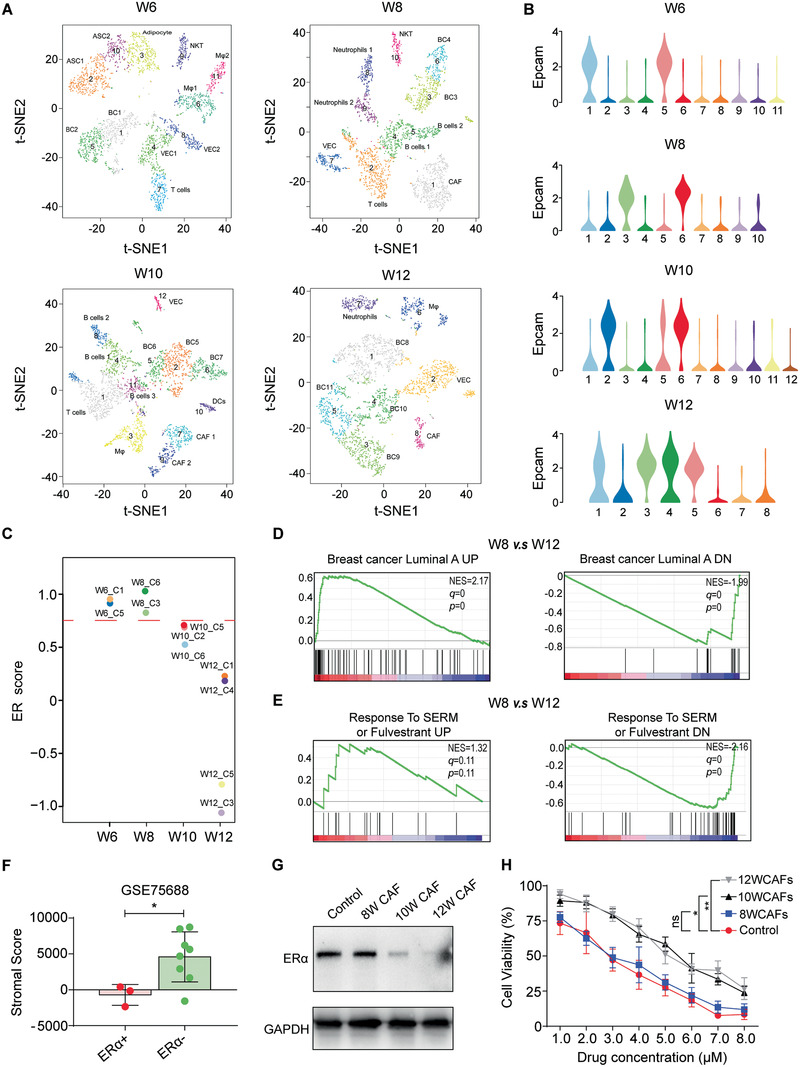
Single‐cell sequencing reveals that CAFs are associated with poor tamoxifen response in breast cancer. A) t‐SNE plot of 3000 cells from primary breast cancer tumors from W6–W12 MMTV‐PyMT mice. K‐means clustering was applied to the t‐SNE plot to identify the major cell types. Clusters are highlighted in different colors. B) Violin plots showing the distribution of Epcam expression in each cluster. Eleven clusters showing high Epcam expression were tumor cells. C) The ER scores of clusters with high Epcam expression were analyzed (R software package genefu^[^ ^19^ ^]^). W6_C1, cluster 1 of W6; W6_C5, cluster 5 of W6; W8_C3, cluster 3 of W8; W8_C6, cluster 6 of W8; W10_C2, cluster 2 of W10; W10_C5, cluster 5 of W10; W10_C6, cluster 6 of W10; W12_C1, cluster 1 of W12; W12_C3, cluster 3 of W12; W12_C4, cluster 4 of W12; W12_C5, cluster 5 of W12. D) GSEA revealed the enrichment of gene sets related to “Luminal A” in the ranked gene list of all BCs from W8 MMTV‐PyMT mice versus all BCs from W12 MMTV‐PyMT mice. E) GSEA revealed the enrichment of gene sets related to “Response to tamoxifen or fulvestrant” in the ranked gene list of all BCs from W8 MMTV‐PyMT mice versus all BCs from W12 MMTV‐PyMT mice. F) The ESTIMATE algorithm^[^ ^57^ ^]^ was used to analyze the stromal score of ER*α*‐positive or ER*α*‐negative human primary breast cancer tissues from the Gene Expression Omnibus (GSE75688). A higher “stromal score” means more CAFs. G) Western blotting was conducted to detect ER*α* expression in ER*α*‐positive BCs derived from W8 MMTV‐PyMT mice (alone or cocultured with CAFs from W8‐W12 MMTV‐PyMT mice). Monocultures of BCs from W8 mice were used as a control. H) Viability of ER*α*‐positive BCs (alone or cocultured with CAFs from W8‐W12 MMTV‐PyMT mice) derived from W8 MMTV‐PyMT mice in the presence of 4‐hydroxytamoxifen. Monocultures of BCs from W8 mice were used as a control. IC50 (control) = 2.12 × 10^−6^ m (95% CI 1.67 to 2.69); IC50 (8WCAFs) = 2.41 × 10^−6^ m (95% CI 2.00 to 2.91); IC50 (10WCAFs) = 5.74 × 10^−6^ m (95% CI 4.56 to 7.21); IC50 (12 WCAFs) = 6.02 × 10^−6^ m (95% CI 4.81 to 7.53); F,H) The data are shown as the means ± S.E.M. ns *p* > 0.05, ^:^ *p* < 0.05, ^::^ *p* < 0.01. F) Unpaired *t*‐test.

To gain greater insights into epithelial ER*α* downregulation and the poor tamoxifen response in breast cancer from the perspective of the TME, we performed single‐cell RNA sequencing (scRNA‐seq) on primary tumors from W6‐W12 MMTV‐PyMT mice. We charted the microenvironment landscape during breast cancer progression in a mouse model with t‐distributed stochastic neighbor embedding (t‐SNE) plots. A list of differentially expressed genes (DEGs) that defined the clusters was presented in Table S1 in the Supporting Information. Upon analysis of the DEGs, we identified 11 major cell types: breast cancer cells (i.e., expressing *Epcam*; BCs), CAFs (i.e., expressing *Col1A1, Col3A1, THY1*, and *FAP*), natural killer T (NKT) cells, T cells, B cells, vascular endothelial cells (VECs), adipocyte stem cells (ASCs), adipocytes, macrophages (M*φ*), dendritic cells and neutrophils (**Figure** **2**A). First, to observe the dynamic changes in BCs during breast cancer progression, we selected all 11 clusters of BCs (Figure 2B) and analyzed the ER scores of the putative BCs using the R software package genefu.^[^ ^19^ ^]^ Consistent with the immunohistochemical results in Figure 1E, BCs from W6 and W8 MMTV‐PyMT mice showed higher ER*α* expression than those from W10 and W12 MMTV‐PyMT mice (Figure 2C). Furthermore, we used the W8 group to represent the group with high ER*α* expression and the W10 and W12 groups to represent the group with low ER*α* expression. The expression profiles of all BCs in each group were integrated. Gene set enrichment analysis (GSEA) showed that the “Luminal A Breast Cancer (ER*α*‐positive breast cancer)” and “Response to tamoxifen or fulvestrant” signatures were enriched in BCs of the W8 group but not of the W10 or W12 groups (Figure 2D,E, Figure S1A,B, Supporting Information).

To gain a more in‐depth understanding of ER*α* downregulation in BCs, we generated a heat map that displayed the ER*α* score and cell type of each group (Figure S1C, Supporting Information). We observed that CAFs were detected in both the W10 and W12 groups, which had low ER*α* expression. These results suggest that CAFs may be involved in inducing ER*α* downregulation in BCs. Moreover, given that CAFs are the most prominent cell type within the tumor stroma, which exists in close proximity to breast cancer cells, we focused on CAFs to be a promising suppressor of ER*α* expression in breast cancer cells. We analyzed the gene expression profiling of bulk ER*α*‐positive and ER*α*‐negative human breast cancer tissues, and the results suggested more CAF infiltration in ER*α*‐negative primary tumors than in ER*α*‐positive primary tumors (Figure 2F). Furthermore, we isolated CAFs from W10 and W12 MMTV‐PyMT mice. By coculturing these CAFs with ER*α*‐positive BCs, we confirmed that CAFs from the W10 or W12 group could significantly induce ER*α* downregulation and tamoxifen resistance (Figure 2G,H). Interestingly, CAFs were also detected in the W8 group but did not have a similar effect on BCs (Figure 2G,H).

### 2.3 CD63^+^ CAFs Induce ER*α* Downregulation and Tamoxifen Resistance

**Figure 3:**
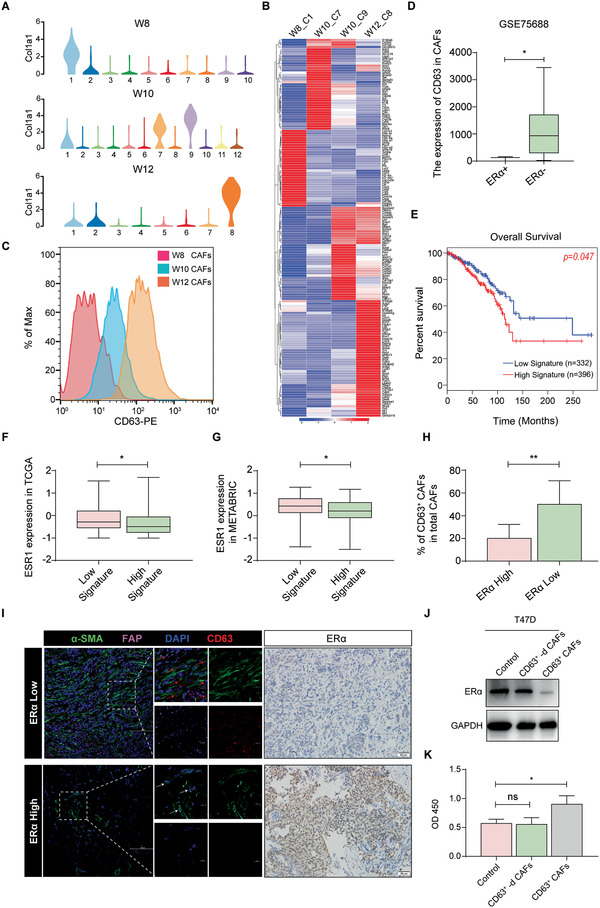
CD63^+^ CAFs induce ER*α* downregulation and tamoxifen resistance. A) Violin plots showing the expression distribution of Col1a1 in each cluster. Cluster 1 of W8, clusters 7 and 9 of W10, and cluster 8 of W12 had high Col1a1 expression. B) Heatmap representing DEGs of the CAFs. C) Flow cytometric analysis of CD63 expression in CAFs isolated from primary tumors of W8‐W12 MMTV‐PyMT mice. D) Analysis of CD63 expression in infiltrating CAFs from ER*α*‐positive or ER*α*‐negative primary breast cancer tissues. The single‐cell sequencing data were from the Gene Expression Omnibus (GSE75688). E) Overall survival (months) analysis of the CD63^+^ CAF gene signature (CD63, Col1a1, Col3a1, Thy1, FAP) in breast cancer patients. F,G) Analysis of ER*α* expression in ER*α*‐positive breast cancer patients based on high or low expression of the CD63‐CAF gene signature (CD63, Col1a1, Col3a1, Thy1, FAP). F) Data from TCGA; low (*n* = 81), high (*n* = 87). G) Data from METABRIC; low (*n* = 80), high (*n* = 79). H) The percentage of CD63^+^ CAFs among the total CAF population. I) Representative images of *α*‐SMA, FAP, and CD63 immunofluorescent staining in human primary breast cancer tissue with high or low ER*α* expression. (*n* = 19). The red arrows indicate CD63^+^ CAFs; the white arrows indicate CD63^−^ CAFs. J) ER*α* expression in T47D cells either cultured alone or cocultured with CD63^+^ CAFs or CD63^+^‐depleted CAFs. CD63^+^ CAFs and CD63^+^‐depleted CAFs were isolated from human primary breast cancer tissue. K) Viability of T47D cells alone or cocultured with CD63^+^ CAFs or CD63^+^‐depleted CAFs in the presence of 4‐hydroxytamoxifen. D,F–H,K) The data are shown as the means ± S.E.M. ns *p* > 0.05, ^:^ *p* < 0.05, ^::^ *p* < 0.01. E) Log‐rank test. F–H) Unpaired *t*‐test. K) ANOVA with Dunnett's *t*‐test.

The above results suggest that CAFs from the W10 or W12 group, but not from the W8 group, induce ER*α* downregulation and tamoxifen resistance. Therefore, we intended to search for cell‐surface markers to distinguish these functionally distinctive CAFs, which might promote live‐cell sorting for CAF subpopulations to investigate their functional heterogeneity and promote the development of effective targeting therapy against cancer‐promoting CAF subsets. Fortunately, by analyzing the molecular signatures of these CAFs with scRNA‐seq (**Figure** **3**A,B; Figure S2A, Supporting Information), we found that the membrane protein CD63 was upregulated in W10 and W12 CAFs compared to W8 CAFs (Figure S2B, Supporting Information). Next, we confirmed by flow cytometry that CD63 was highly expressed in W10 and W12 CAFs (Figure 3C). To evaluate the expression of the membrane protein CD63 in human CAFs, we reviewed publicly available scRNA‐seq data from human primary breast cancer and found that CAFs infiltrating ER*α*‐negative breast cancer tissue showed significantly higher CD63 expression than those infiltrating ER*α*‐positive breast cancer tissue (Figure 3D). These results indicate that CD63 may be a cell‐surface marker distinguishing CAFs which can induce tamoxifen resistance.

To further explore the clinical significance of CD63^+^ CAFs in breast cancer, we generated a gene signature to evaluate the abundance of CD63^+^ CAFs in primary breast tumors and performed an analysis with the TCGA dataset. The results showed that the high CD63^+^ CAF gene signature group had a worse prognosis than the low CD63^+^ CAF gene signature group (Figure 3E). Additionally, we found that ER*α* expression was significantly higher in the low CD63^+^ CAF gene signature group than in the high CD63^+^ CAF gene signature group (Figure 3F,G). We also evaluated the prevalence of CD63^+^ CAFs in human breast cancer tissues by confocal microscopy. The results showed that CD63^+^ CAFs were more abundant in tissues with low ER*α* expression than in tissues with high ER*α* expression (Figure 3H,I). More importantly, we isolated CD63^+^ CAFs and CD63^+^‐depleted CAFs (Figure S2C, Supporting Information) and cocultured them with ER*α*‐positive BCs. CD63^+^ CAFs but not CD63^+^‐depleted CAFs could induce ER*α* downregulation and tamoxifen resistance (Figure 3J,K; Figure S2D–G, Supporting Information).

### 2.4 CD63^+^ CAF‐Derived Exosomal miR‐22 Promotes Tamoxifen Resistance

**Figure 4:**
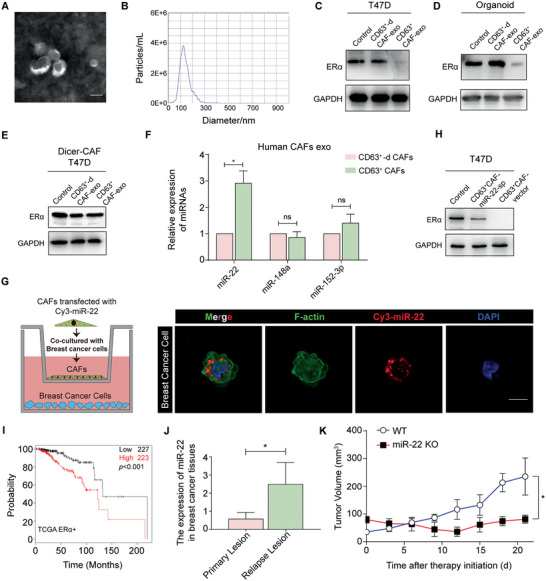
CD63^+^ CAF‐derived exosomal miR‐22 promotes tamoxifen resistance. A) Representative transmission electron microscopy images of exosomes derived from CAFs (scale bar, 100 nm). B) Nanoparticle tracking analysis shows the size distribution of CAF‐derived exosomes. ER*α* expression in C) T47D cells and D) breast cancer organoids treated with vehicle, CD63^+^ CAFs or CD63^+^‐depleted CAF‐derived exosomes. E) ER*α* expression in T47D cells treated with vehicle, Dicer‐knockdown CD63^+^ CAFs or Dicer‐knockdown CD63^+^‐depleted CAF‐derived exosomes. F) The miR‐22, miR‐152‐3p and miR‐148a expression levels in CD63^+^ CAFs or CD63^+^‐depleted CAF‐derived exosomes were determined using real‐time PCR. G) CAFs transiently transfected with Cy3‐tagged miR‐22 (Cy3‐miR‐22) were cocultured with BCs for 48 h. Representative confocal images of Cy3‐miR‐22 (red) and F‐actin (green) immunostaining (scale bar, 10 µm). H) ER*α* expression in T47D cells. Control, treated with vehicle; CD63^+^CAF‐miR‐22‐sp, treated with exosomes derived from CD63^+^ CAFs with miR‐22 knockdown; CD63^+^CAF‐vector treated with exosomes derived from control CD63^+^ CAFs. I) Overall survival (months) analysis of miR‐22 in ER*α*‐positive breast cancer patients. Data from TCGA. J) miR‐22 expression in paired primary and recurrent lesions of breast cancer patients receiving tamoxifen therapy. The data were from Gene Expression Omnibus (GSE83292). K) Average volume of tumors from 9‐week‐old MMTV‐PyMT+Mir22−/− mice and MMTV‐PyMT+Mir22+/+ mice. Mice were treated with tamoxifen (*n* = 5). The data are shown as the means ± S.E.M. ^:^ *p* < 0.05. I) Log‐rank test. F,J) Paired *t*‐test. K) Unpaired *t*‐test.

Exosomes are nanometric membrane vesicles that play an important role in intracellular communication.^[^ ^20^, ^21^ ^]^ Therefore, we investigated whether CAF‐derived exosomes might induce ER*α* downregulation. First, we isolated and purified exosomes from the conditioned medium of CAFs via the standard exosome isolation method of ultracentrifugation. The cup‐shaped structure, size, and number of the isolated exosomes were identified by electron microscopy and NanoSight particle tracking analysis (**Figure** **4**A,B). Exosome markers were detected by Western blotting analysis (Figure S3A, Supporting Information). Then, we treated ER*α*‐positive BCs or breast cancer organoids with CAF‐derived exosomes and observed that CD63^+^ CAF‐derived exosomes could induce ER*α* downregulation (Figure 4C,D; Figure S3B,C, Supporting Information).

It has been reported that microRNAs (miRNAs) are the most abundant macromolecules in exosomes, and exosome‐mediated miRNA delivery is widely believed to contribute to drug resistance in many cancers.^[^ ^22^, ^23^, ^24^ ^]^ Therefore, we investigated whether CD63^+^ CAF‐derived exosomal miRNAs could induce ER*α* downregulation in BCs. First, we knocked down Dicer, a protein essential for the biogenesis of miRNAs, in CD63^+^ CAFs and CD63^+^‐depleted CAFs. Then, we treated ER*α*‐positive BCs with exosomes derived from Dicer‐knockdown CAFs and observed that these exosomes could not induce ER*α* downregulation (Figure 4E; Figure S3D, Supporting Information). These results suggest that CD63^+^ CAFs suppress ER*α* expression in BCs mainly via exosomal miRNAs.

To identify the specific miRNAs involved, the miRNA expression levels in exosomes derived from CD63^+^ CAFs and CD63^+^‐depleted CAFs were analyzed via miRNA‐seq, and the top miRNAs (fold change > 2) were selected (Table S2, Supporting Information). On this basis, we performed an analysis using TargetScan to identify miRNAs that might conservatively target the 3′UTR of human ER*α* mRNA and found three candidates (miR‐22, miR‐148a, miR‐152‐3p). We then confirmed that miR‐22 was the most highly enriched miRNA in CD63^+^ CAF‐derived exosomes (Figure 4F; Figure S3E, Supporting Information). Next, we observed that BCs expressed higher levels of miR‐22 when cocultured with CD63^+^ CAFs; however, miR‐22 expression in BCs was substantially reduced when CD63^+^ CAF‐derived exosomes were pharmacologically depleted (Figure S3F, Supporting Information). Additionally, we confirmed by confocal microscopy that miR‐22 could be transferred from CAFs to BCs (Figure 4G). These results suggest that miR‐22 is transferred from CD63^+^ CAFs to BCs via exosomes. We knocked down miR‐22 expression in CD63^+^ CAFs using a miRNA sponge and observed that decreased miR‐22 expression in CD63^+^ CAF exosomes compromised its suppressive effect on ER*α* and ability to induce tamoxifen resistance (Figure 4H; Figure S3G,H, Supporting Information).

To further investigate the effect of miR‐22 on tamoxifen resistance, we analyzed patient data from a public database and found that in ER*α*‐positive (tamoxifen‐sensitive) breast cancer patients, the group with high miR‐22 expression had a poorer prognosis than the group with low miR‐22 (Figure 4I; Figure S3I, Supporting Information). Then, we analyzed miR‐22 expression in paired primary and recurrent lesions of breast cancer patients receiving tamoxifen therapy. The results showed that miR‐22 expression was significantly higher in the recurrent lesions than in the matched primary lesions (Figure 4J). Moreover, tamoxifen treatment showed that the MMTV‐PyMT+Mir22−/− mice were more sensitive to tamoxifen than the MMTV‐PyMT+Mir22+/+ mice (Figure 4K). BCs transfected with miR‐22 mimics were less sensitive to tamoxifen than the control BCs (Figure S3J, Supporting Information). Collectively, these results indicate that miR‐22, which is enriched in CD63^+^ CAF‐derived exosomes, mediates tamoxifen resistance in breast cancer.

### 2.5 ESR1 and PTEN are Direct Targets of Exosomal miR‐22 in Breast Cancer Cells

**Figure 5:**
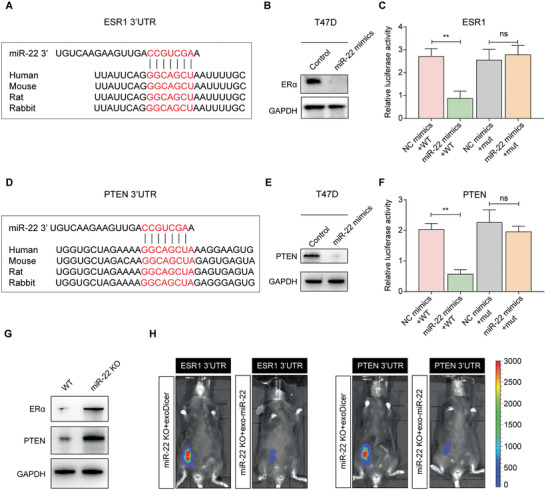
ESR1 and PTEN are direct targets of exosomal miR‐22 in breast cancer cells. A) Predicted binding site of miR‐22 on the ESR1 3′UTR; highly conserved binding site (red) across different species. B) Western blotting was conducted to detect ER*α* expression in BCs transfected with control mimics or miR‐22 mimics. C) Effects of miR‐22 on luciferase activity of the reporter gene bearing wild‐type (WT) or mutant (mut) 3′UTRs of ESR1 in HEK‐293T cells. mut: the core sequence was mutated to “UUACUAG.” D) Predicted binding site of miR‐22 to the PTEN 3′UTR; highly conserved binding site (red) across different species. E) Western blotting was conducted to detect PTEN expression in BCs transfected with control mimics or miR‐22 mimics. F) Effects of miR‐22 on the luciferase activity of the reporter gene bearing wild‐type (WT) or mutant (mut) 3′UTRs of PTEN in HEK‐293T cells. mut: the core sequence was mutated to “UUACUAGC.” G) Western blotting was conducted to detect the expression of ER*α* and PTEN in BCs derived from MMTV‐PyMT+Mir22−/− or MMTV‐PyMT+Mir22+/+ mice. H) Representative images of miR‐22 KO mice that were orthotopically transduced with either pacAd5‐Luc‐ESR1‐3′UTR or pacAd5‐Luc‐PTEN‐3′UTR luciferase reporter,^[^ ^58^ ^]^ i.v. injected with exosomes derived from either Dicer‐knockdown or miR‐22‐overexpressing CD63^+^ CAFs, and subjected to IVIS analysis (*n* = 5). C,F) The data are shown as the means ± S.E.M. ns *p* > 0.05. ^::^ *p* < 0.01. ANOVA with Tukey's *t*‐test.

To delineate the molecular mechanisms underlying the role of exosomal miR‐22 in conferring tamoxifen resistance in breast cancer cells, we used bioinformatics tools (TargetScan) to predict evolutionarily conserved target genes. First, ESR1 was verified to be a direct target of miR‐22 (**Figure** **5**A), and Western blotting assays revealed that BCs transfected with miR‐22 mimics showed lower ER*α* expression than control BCs (Figure 5B). Subsequently, the reporter gene assay showed that the effect of miR‐22 on luciferase activity was abrogated when cells were transfected with mutant 3′UTRs of ESR1 (Figure 5C). Our finding is consistent with previous studies that demonstrate miR‐22 as a strong ER*α* repressor.^[^ ^25^ ^]^

We also found that PTEN was a direct target of miR‐22 (Figure 5D). A large body of experimental and clinical evidence has determined that loss of PTEN promotes tamoxifen resistance in breast cancer.^[^ ^26^, ^27^ ^]^ Moreover, loss of PTEN in ER*α*‐positive breast cancer is predictive of reduced recurrence‐free survival after tamoxifen.^[^ ^28^ ^]^ Western blotting assays revealed that BCs transfected with miR‐22 mimics showed lower PTEN expression than control BCs (Figure 5E). Subsequently, the reporter gene assay showed that the effect of miR‐22 on luciferase activity was abrogated when cells were transfected with mutant 3′UTRs of PTEN (Figure 5F). Additionally, BCs were isolated from MMTV‐PyMT+Mir22−/− mice (miR‐22 KO) or MMTV‐PyMT+Mir22+/+ mice (WT). Western blotting assays showed that the expression levels of ER*α* and PTEN in BCs from the miR‐22 KO group were higher than those from the WT group (Figure 5G). Then, the data from the public database confirmed that the transfection of miR‐22 mimics into BCs induced the downregulation of ER*α* and PTEN mRNA (Figure S4A,B, Supporting Information).

To test whether exosomal miR‐22 could regulate ESR1 and PTEN in vivo, we first transduced miR‐22 KO mice with a pacAd5‐Luc‐ESR1‐3′UTR or pacAd5‐Luc‐PTEN‐3′UTR luciferase reporter and measured the levels of ESR1 and PTEN expression after the injection of different CD63^+^ CAF‐derived exosomes. The injection of exosomes from miR‐22‐overexpressing CD63^+^ CAFs into miR‐22 KO mice suppressed the activities of both the ESR1 and PTEN reporters (Figure 5H, Figure S4C,D, Supporting Information). Collectively, these results suggest that CD63^+^ CAF‐derived exosomal miR‐22 could suppress ER*α* and PTEN expression in BCs.

### 2.6 Prolonged STAT3 Activation Maintains the Phenotypes and Functions of CD63^+^ CAFs

**Figure 6:**
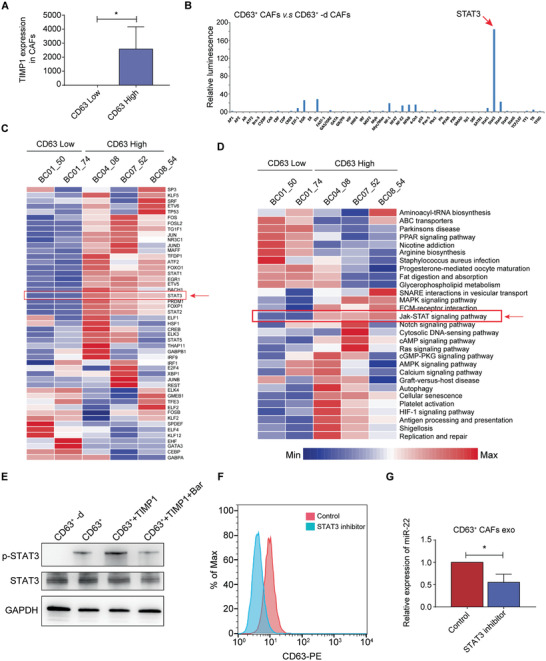
Prolonged STAT3 activation maintains the phenotypes and functions of CD63^+^ CAFs. A,C,D) Human primary breast cancer scRNA‐seq data were obtained from the gene expression omnibus (GSE75688). BC04_08, BC07_52 and BC08_54 were CAFs with high CD63 expression, and BC01_50 and BC01_74 were CAFs with low CD63 expression. A) Single‐cell sequencing revealed TIMP1 expression in CAFs with high or low CD63 expression. C) Heatmap of the *t* values of AUC scores of TF‐mediated regulation of expression, as estimated using SCENIC.^[^ ^59^ ^]^ D) Differences in pathway activities scored per cell by GSVA between CAFs with high and those with low CD63 expression. B) The TF Activation Profiling Plate Array Assay acquired RLUs of CD63^+^ CAFs or CD63^+^‐depleted CAFs, showing the 48 TF activation states. E) Western blotting was conducted to detect the expression of STAT3 and the levels of p‐STAT3 in different CAFs. CD63^+^‐d: CD63^+^‐depleted CAFs, CD63^+^: CD63^+^ CAFs in the presence of blocking antibodies against TIMP1, CD63^+^+TIMP1: CD63^+^ CAFs treated with blocking antibodies against TIMP1 followed by treatment with 5 ng mL^−1^ human recombinant TIMP1, CD63^+^+TIMP1+Bar: CD63^+^ CAFs treated with blocking antibodies against TIMP1 followed by treatment with 5 ng mL^−1^ human recombinant TIMP1 and the JAK inhibitor baricitinib. F) Flow cytometric analysis of CD63 expression in control CD63^+^ CAFs and CD63^+^ CAFs treated with a STAT3 inhibitor. G) miR‐22 expression in exosomes from control CD63^+^ CAFs and STAT3 inhibitor‐treated CD63^+^ CAFs was determined using real‐time PCR. A,G) The data are shown as the means ± S.E.M. ^:^ *p* < 0.05. A) Unpaired *t*‐test. G) Paired *t*‐test.

To elucidate the molecular mechanism involved in sustaining the phenotype and function of CD63^+^ CAFs, we first reanalyzed the scRNA‐seq data of CAFs (Figure 3A,B). Interestingly, we observed that TIMP1 was upregulated in CAFs with high CD63 expression (Figure S5A, Supporting Information). By analyzing the publicly available scRNA‐seq data of human primary breast cancer, we confirmed that TIMP1 expression in CAFs with high CD63 expression was significantly higher than in CAFs with low CD63 expression (**Figure** **6**A). TIMP1 is a known ligand of CD63.^[^ ^29^, ^30^ ^]^ To assess which transcription factors (TFs) differ between these CAFs, we isolated CD63^+^ CAFs and CD63^+^‐depleted CAFs and performed a TF activation profiling plate array assay. We observed that STAT3 was dramatically activated with the largest fold change in CD63^+^ CAFs (Figure 6B). Then, we applied single‐cell regulatory network inference and clustering (SCENIC) to infer the activity of TFs based on the expression of their putative target genes. We also observed that genes regulated by STAT3 were highly upregulated in CAFs with high CD63 expression (Figure 6C). These results suggest that STAT3 is the key factor for CD63^+^ CAFs. Furthermore, we analyzed the gene signature score of activated or tyrosine‐phosphorylated STAT3 (pSTAT3)^[^ ^31^ ^]^ and confirmed that STAT3 was highly activated in CAFs with high CD63 expression (Figure S5B, Supporting Information). Subsequently, to further elucidate the signaling pathways that participate in STAT3 activation, we compared pathway activities by gene set variation analysis (GSVA) and found that the Jak‐STAT signaling pathway was highly activated in CAFs with high CD63 expression (Figure 6D). Therefore, we speculated that TIMP1 might induce STAT3 activation through the Jak‐STAT signaling pathway. To verify these findings, we treated CD63^+^ CAFs with human recombinant TIMP1 and observed that recombinant TIMP1 could induce STAT3 activation, while treatment with a Jak‐STAT signaling pathway inhibitor reversed this effect (Figure 6E). Next, we generated different STAT3 reporter‐CAFs with stable expression of the STAT3 response element‐driven luciferase reporter.^[^ ^32^ ^]^ Similarly, the results showed that recombinant TIMP1 could promote the transcriptional activity of STAT3, while treatment with a Jak‐STAT signaling pathway inhibitor reversed this effect (Figure S5C, Supporting Information). The above results indicate that the binding of TIMP1 to CD63 induced STAT3 activation in CD63^+^ CAFs mainly via the Jak‐STAT signaling pathway.

We next investigated how STAT3 activation in CD63^+^ CAFs can sustain the phenotype of CD63^+^ CAFs. We found a potential STAT3 binding site in the promoter region of CD63 (Figure S5D, Supporting Information) and performed chromatin immunoprecipitation (ChIP) assays in CD63^+^ CAFs and CD63^+^‐depleted CAFs. The results showed that the STAT3 antibody pulled down higher amounts of CD63 promoter DNA in CD63^+^ CAFs than in CD63^+^‐depleted CAFs (Figure S5E, Supporting Information). Moreover, flow cytometry showed that the STAT3 inhibitor induced the downregulation of CD63 expression on the cell surface of CD63^+^ CAFs (Figure 6F). Then we treated CD63^+^ CAFs with human recombinant TIMP1 and observed that recombinant TIMP1 could promote CD63 expression on the cell surface of CD63^+^ CAFs, while treatment with a STAT3 inhibitor reversed this effect (Figure S5F, Supporting Information).

Finally, we investigated how STAT3 activation in CD63^+^ CAFs can sustain the function of CD63^+^ CAFs. We identified a potential STAT3 binding site in the promoter region of miR‐22 (Figure S5D, Supporting Information). The ChIP assay showed that the STAT3 antibody pulled down higher amounts of miR‐22 promoter DNA in CD63^+^ CAFs than in CD63^+^‐depleted CAFs (Figure S5G, Supporting Information). Moreover, real‐time PCR showed that the STAT3 inhibitor significantly suppressed the level of miR‐22 expression in both CD63^+^ CAFs and CD63^+^ CAF‐derived exosomes (Figure 6G; Figure S5H, Supporting Information). Then we treated CD63^+^ CAFs with human recombinant TIMP1 and observed that recombinant TIMP1 could promote miR‐22 expression in both CD63^+^ CAFs and CD63^+^ CAF‐derived exosomes, while treatment with a STAT3 inhibitor reversed this effect (Figure S5I,J, Supporting Information). Collectively, these results suggest that the binding of TIMP1 to CD63 on the cell surface sustains the expression of CD63 and miR‐22 in CD63^+^ CAFs via STAT3 activation mainly through the Jak‐STAT signaling pathway.

### 2.7 The SFRS1 Protein Mediates miR‐22 Packaging into CD63^+^ CAF‐Derived Exosomes

**Figure 7:**
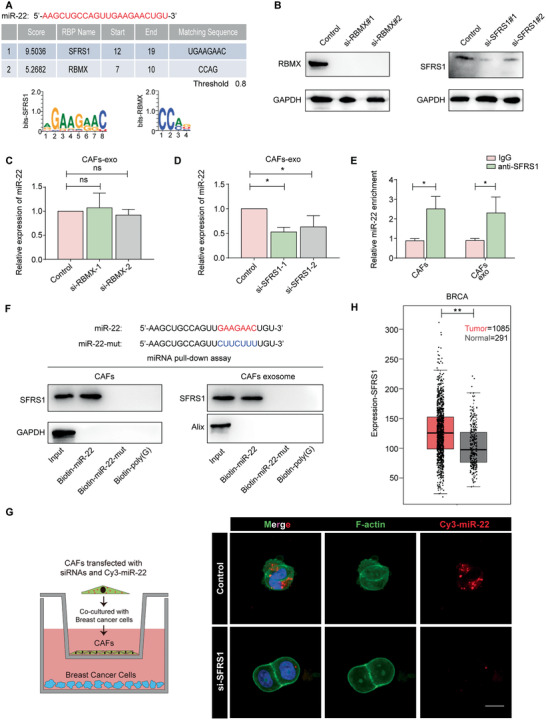
The SFRS1 protein mediates miR‐22 packaging into CD63^+^ CAF‐derived exosomes. A) A specific interaction between the miR‐22 sequence and RBP motifs was predicted via RBPDB analysis (threshold 0.8).^[^ ^40^, ^60^ ^]^ B) Western blotting results showing RBMX and SFRS1 expression levels in CD63^+^ CAFs at 48 h after transfection with specific siRNAs. C,D) miR‐22 expression in exosomes derived from CD63^+^ CAFs transfected with specific siRNAs targeting RBMX or SFRS1 was measured using real‐time PCR. E) RIP assays with anti‐SFRS1 antibody (or IgG as negative control) were performed on the cell and exosomal lysates from CD63^+^ CAFs. miR‐22 levels in immunoprecipitated samples were normalized to the corresponding input sample. F) Western blot analysis of SFRS1 expression in cell and exosomal lysates from CD63^+^ CAFs subjected to miRNA pulldown with biotinylated miR‐22 or biotinylated miR‐22 mutant; biotinylated poly(G) was used as a negative control. G) BCs were cocultured with CAFs transfected with Cy3‐miR‐22 and specific siRNAs targeting SFRS1 for 48 h. Representative confocal images of Cy3‐miR‐22 (red) and F‐actin (green) immunostaining (scale bar, 10 µm). H) The SFRS1 mRNA expression level in primary breast cancer tissues (*n* = 1085) and adjacent normal tissues (*n* = 291). Data from the TCGA dataset. C,D,E,H) The data are shown as the means ± S.E.M. ns *p* > 0.05. ^:^ *p* < 0.05. ^::^ *p* < 0.01. C,D) ANOVA with Dunnett's *t*‐test. E–H) Unpaired *t*‐test.

To investigate how miR‐22 in CD63^+^ CAFs was packaged into exosomes, we analyzed the specific interactions between the miR‐22 sequence and RNA‐binding protein (RBP) motifs. The results revealed that SFRS1 and RBMX had miR‐22‐specific binding sites (**Figure** **7**A), and further investigations showed that only SFRS1 knockdown with specific siRNAs in CD63^+^ CAFs significantly decreased the level of miR‐22 in the released exosomes (Figure 7B–D). This indicated that the exosomal sorting of miR‐22 was highly dependent on SFRS1. Moreover, RNA immunoprecipitation (RIP) assays with cell and exosome lysates from CD63^+^ CAFs were performed, and the results showed that miR‐22 was enriched in the SFRS1 antibody group compared with the IgG group (Figure 7E). Additionally, miRNA pull‐down assays revealed an interaction between SFRS1 and miR‐22 in both CD63^+^ CAFs and CD63^+^ CAF‐derived exosomes (Figure 7F). However, the binding of SFRS1 to miR‐22 was impaired when the core interaction sequence (GAAGAAC) was mutated (Figure 7F). Then, confocal microscopy revealed that the transport capacity of miR‐22 from CAFs to BCs was impaired when CAFs were transfected with SFRS1‐specific siRNAs (Figure 7G). Moreover, we found that SFRS1 expression was upregulated in breast cancer tissues compared with adjacent normal tissues (Figure 7H). These results revealed that SFRS1 was a key factor in packaging miR‐22 into exosomes through binding a specific motif (GAAGAAC) of miR‐22.

### 2.8 Pharmacological Inhibition of CD63^+^ CAF Activity Sensitizes Breast Tumors to Tamoxifen Therapy

**Figure 8:**
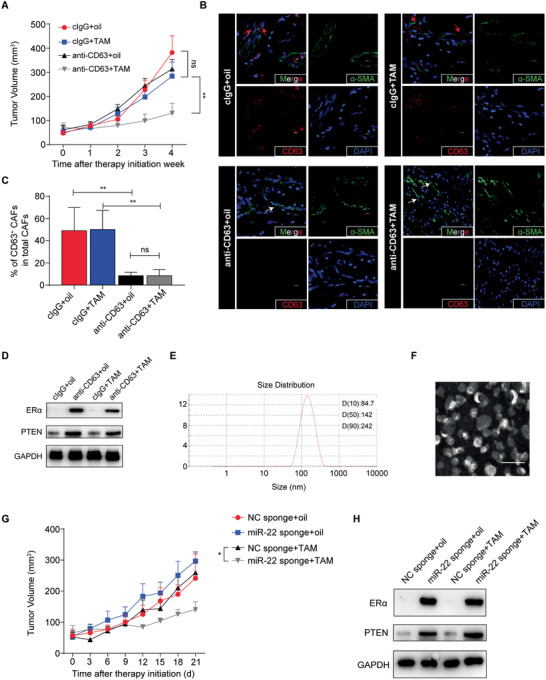
Pharmacological inhibition of CD63^+^ CAF activity sensitizes breast tumors to tamoxifen therapy. ER*α*‐positive breast cancer cells mixed with CD63^+^ CAFs were implanted into nude mice, which were then coadministered with tamoxifen and anti‐CD63 neutralizing antibody. Corn oil and a nonspecific IgG1 isotype control antibody were used as the respective negative controls. A) Tumor size was monitored for 4 weeks (*n* = 5). B) Representative images of *α*‐SMA and CD63 immunofluorescent staining in breast cancer tissue. Scale bars, 10 µm. The red arrows indicate CD63^+^ CAFs; the white arrows indicate CD63^−^ CAFs. C) The percentage of CD63^+^ CAFs in the total CAF population. Related to (B). D) BCs were isolated from the harvested breast cancer tissue. The expression of ER*α* and PTEN in these cells was determined by Western blotting. E) Size distribution of cRGD‐decorated nanoparticles as measured by dynamic light scattering analysis. F) Transmission electron microscopy image of cRGD‐decorated nanoparticles. Scale bars, 200 nm. G) A combination of tamoxifen and cRGD‐miR‐22‐sponge nanoparticles was administered to MMTV‐PyMT mice, with corn oil and cRGD‐NC‐sponge nanoparticles as the respective negative controls. The average volume of tumors from 9‐week‐old MMTV‐PyMT mice was measured (*n* = 5). H) BCs were isolated from the harvested breast cancer tissue. The expression of ER*α* and PTEN in cancer cells was determined by Western blotting. A,C,G) The data are shown as the means ± S.E.M. ns *p* > 0.05. ^:^ *p* < 0.05. ^::^ *p* < 0.01. A,C,G) ANOVA with Tukey's *t*‐test.

To further investigate whether inhibiting CD63^+^ CAF activity could sensitize breast tumors to tamoxifen therapy, we intraperitoneally administered anti‐CD63 neutralizing monoclonal antibody to tumor‐bearing mice, and the results showed that it significantly enhanced the effectiveness of tamoxifen treatment (**Figure** **8**A). Confocal microscopy showed that the number of CD63^+^ CAFs was decreased in the anti‐CD63 neutralizing antibody treatment group (Figure 8B,C). Then we isolated exosomes from the serum of the mice and performed real‐time PCR. The results showed that circulating exosomal miR‐22 was decreased in the anti‐CD63 neutralizing antibody treatment group (Figure S6A, Supporting Information). In addition, BCs from the anti‐CD63 neutralizing antibody treatment group expressed higher levels of ER*α* and PTEN than those from the isotype IgG1 treatment group (Figure 8D). Since PTEN and ER*α* are evolutionarily conserved target genes of miR‐22, the above results indicate that the anti‐CD63 antibody could enhance the therapeutic effect of tamoxifen by reducing the infiltration of CD63^+^ CAFs and thus inhibiting the accumulation of CD63^+^ CAF‐derived exosomal miR‐22 in BCs.

Cyclic RGD (cRGD) is a “tumor‐homing” cyclic peptide that binds directly to *αβ* integrin.^[^ ^33^ ^]^ To investigate the therapeutic potential of suppressing miR‐22 expression/function in breast cancer, we generated cRGD‐decorated nanoparticles encapsulating the miR‐22 sponge as a targeted delivery system to cancer cells (Figure 8E,F). Firstly, we confirmed that the cRGD‐decorated nanoparticles could be taken up by the BCs (Figure S6B, Supporting Information). Then we found that in cRGD‐NC‐sponge nanoparticles treated BCs, CD63^+^ CAF‐derived exosomes could suppress PTEN and ER*α* expression (Figure S6C,D, Supporting Information). However, the effect of CD63^+^ CAF‐derived exosomes on PTEN or ER*α* expression was abrogated when cells were treated with cRGD‐miR‐22‐sponge nanoparticles (Figure S6C,D, Supporting Information). These in vitro results indicate that cRGD‐miR‐22‐sponge nanoparticles could suppress CD63^+^ CAFs activity by sequestrating miR‐22, especially exosome‐derived miR‐22, in BCs.

We then tested the cRGD‐miR‐22‐sponge nanoparticles in tumor mouse models. The results showed that nanoparticles carrying the miR‐22 sponge significantly enhanced the effectiveness of tamoxifen treatment (Figure 8G), and BCs from the cRGD‐miR‐22‐sponge nanoparticle treatment group expressed higher levels of ER*α* and PTEN than did BCs from the cRGD‐NC‐sponge nanoparticle treatment group (Figure 8H). These results suggested that miR‐22‐sponge mediated sequestration of miR‐22 in BCs, especially exosome‐derived miR‐22, was responsible for the increased therapeutic effect of tamoxifen.

## 3 Discussion

Understanding the molecular events that confer tamoxifen resistance on ER*α*‐positive breast cancer is of major scientific and therapeutic importance. Tumors are multicellular “organs,” and the surrounding microenvironment can create a dynamic signaling circuitry that nourishes and supports tumor cells, allowing them to develop resistance.^[^ ^7^ ^]^ Recently, TME‐targeted strategies have shown great potential in preventing the acquisition of drug resistance.^[^ ^34^, ^35^, ^36^ ^]^ Therefore, we realized that deciphering the mechanism of tamoxifen resistance from the perspective of the TME might effectively overcome this problem. In this study, we observed dynamic changes in the breast cancer TME using scRNA‐seq and found that CAFs, a major component of the TME, were key factors in inducing ER*α* downregulation and tamoxifen resistance. This result was also consistent with the findings of Roswall et al.^[^ ^37^ ^]^ However, we further confirmed that not all CAFs could induce tamoxifen resistance and that CD63^+^ CAFs, a newly identified CAF subset in the TME, specifically induced tamoxifen resistance. Heterogeneity exists among almost all cell types, especially CAFs, in the TME. Therefore, accurately determining the phenotypic heterogeneity and functional diversity of each kind of cell in the TME and translating these discoveries into benefits for patients are major goals in modern oncology. As mentioned, our findings reveal a new subset of CAFs that induce tamoxifen resistance, which highlights a potential approach to predict the therapeutic efficacy of tamoxifen. Similarly, a recent study identified a unique protumorigenic CAF subset that expressed CD10 and GPR77 and is involved in cancer stemness and chemoresistance.^[^ ^8^ ^]^ Together, these studies suggest that the characterization of different CAF subclones based on specific molecular characteristics can not only deepen our insight into the heterogeneity of CAFs but also guide the development of CAF‐targeting precision therapies and mitigate therapeutic resistance.

Exosomes, particles measuring from 30 to 150 nm in diameter, are critical messengers in intercellular communication.^[^ ^38^, ^39^ ^]^ Recent studies have shown that CAFs are one of the main sources of exosomes in the TME and that CAF‐derived exosomes play an important role in mediating drug resistance.^[^ ^40^, ^41^ ^]^ Yeung and co‐workers reported that CAF‐derived exosomal miR‐21 confers paclitaxel resistance on ovarian cancer cells by targeting APAF1.^[^ ^42^ ^]^ However, few studies have evaluated the role of CAF‐derived exosomes in tamoxifen resistance in breast cancer. Here, we found that CD63^+^ CAFs conferred tamoxifen resistance on BCs via exosomal transfer of miR‐22, which suppressed ER*α* and PTEN expression in BCs. Another issue we addressed is how the highly expressed miR‐22 in CD63^+^ CAFs was packed into exosomes. RBPs such as hnRNPA1 and hnRNPA2B1 have been shown to be involved in exosomal miRNA or lncRNA export by binding specific motifs.^[^ ^43^ ^]^ In this study, we also observed that SFRS1, an RBP, specifically interacted with a particular sequence (GAAGAAC) in miR‐22 to mediate its exosomal sorting. Additional functional factors present in exosomes may also contribute to the exosomal sorting of miR‐22. Therefore, further studies are still needed to fully elucidate the mechanism of how miR‐22 is packaged into exosomes.

Downregulation of ER*α* and overactivation of the PI3K‐AKT pathway are the main mechanisms responsible for tamoxifen resistance.^[^ ^44^, ^45^, ^46^, ^47^ ^]^ ER*α* is the target of tamoxifen, and BCs that lose ER*α* expression are undoubtedly less responsive to tamoxifen therapy.^[^ ^37^, ^48^ ^]^ In addition, activation of the PI3K‐AKT pathway induces tamoxifen resistance mainly through the following aspects: 1) the PI3K‐AKT pathway can lead to a decrease in ER*α* expression,^[^ ^27^, ^49^ ^]^ and 2) the PI3K‐AKT pathway promotes the expression of a series of genes involved in cell proliferation, which allows cells to escape the inhibitory effects of tamoxifen.^[^ ^3^ ^]^ Our study first confirmed the dual function of miR‐22 in breast cancer tamoxifen resistance: the accumulation of miR‐22 in BCs not only results in ER*α* downregulation but also in PI3K‐AKT pathway activation via the downregulation of PTEN, a major negative regulator of the PI3K‐Akt pathway. The key issue was to determine whether suppressing the accumulation of miR‐22 in BCs could improve the therapeutic effect of tamoxifen. For this purpose, tumor‐targeted nanoparticles carrying a miR‐22 sponge were administered tamoxifen in tumor mouse models. This agent promoted higher ER*α* and PTEN expression in tumor tissue and significantly improved the therapeutic effect of tamoxifen. Additionally, Xiong et al. reported that inhibiting endogenous miR‐22 in ER*α*‐negative MDA‐MB‐231 cells could restore the expression of ER*α*.^[^ ^50^ ^]^ Therefore, these findings collectively suggest that miR‐22 might potentially predict the tamoxifen response and serve as a therapeutic target for sensitizing BCs to tamoxifen.

Another key finding of our study is the molecular mechanism by which CD63^+^ CAFs sustain their phenotype and activity in the TME. We found that TIMP1, a well‐known ligand of CD63, was highly expressed in CD63^+^ CAFs and can, by binding to CD63, induce STAT3 activation mainly via the Jak‐STAT signaling pathway in CD63^+^ CAFs. Prolonged STAT3 activation mediates the following effects: 1) further promotion of CD63 expression, which results in the formation of more CD63^+^ CAFs in the TME and continued STAT3 activation, and 2) promotion of the expression of the functional molecule miR‐22 in CD63^+^ CAFs, which is then sorted to exosomes via SFRS1. Therefore, our findings indicate that STAT3 is the key factor for CD63^+^ CAFs. Aberrantly elevated STAT3 activity has been estimated to occur in >70% of human cancers ^[^ ^51^ ^]^ and can promote tumor cell proliferation, invasion, angiogenesis, and resistance to conventional chemotherapy, and radiation therapy.^[^ ^52^ ^]^ To date, several STAT3 inhibitors have shown satisfactory therapeutic antitumor effects in preclinical studies and are in active clinical trials. ^[^ ^53^, ^54^ ^]^ Our study provided evidence that STAT3 exerts another key effect on promoting tamoxifen resistance from the perspective of the breast cancer TME. This point might expand the application of STAT3 inhibitors in the clinical treatment of ER*α*‐positive breast cancer patients.

Our study indicates that CD63^+^ CAFs in the TME constitute a survival niche for BCs that can protect them from tamoxifen during cancer progression, which suggests that CD63^+^ CAFs may serve as a novel therapeutic target to enhance tamoxifen sensitivity in breast cancer. In this context, an anti‐CD63 neutralizing monoclonal antibody was administered with tamoxifen in tumor mouse models. The anti‐CD63 neutralizing monoclonal antibody could reduce the infiltration of CD63^+^ CAFs and significantly improve the therapeutic effect of tamoxifen. Moreover, it has been reported that cell surface protein‐CD63‐positive BCs have higher invasive ability and are resistant to chemotherapy.^[^ ^55^ ^]^ These findings collectively highlight the therapeutic potential of a neutralizing monoclonal antibody against CD63, as the antibody could not only successfully eradicate CD63^+^ CAFs and thus improve tamoxifen efficacy in tumor‐bearing mice but also suppress breast cancer metastasis and improve the therapeutic effect of chemotherapy by eradicating cell surface protein‐CD63‐positive BCs.

Overall, these results showed that CD63^+^ CAFs could promote tamoxifen resistance through exosomal miR‐22, which induced downregulation of ER*α* and PTEN expression in BCs. Future comprehensive intervention measures that target every aspect of CD63^+^ CAF activity from CD63^+^ CAFs to exosomal miR‐22 hold promising therapeutic potential to enhance tamoxifen sensitivity and further improve the outcomes of ER*α*‐positive breast cancer patients.

## 4 Experimental Section

##### Antibodies and Inhibitors

The antibodies and dilutions used were as follows: ER*α* (ab32063: immunohistochemistry, 1:150; immunoblotting, 1:750) from Abcam; GAPDH (CW0101: immunoblotting, 1:1000) from CWBIOTECH; PTEN (ab170941: immunoblotting, 1:1000) from Abcam; SFRS1 (12929‐2‐ap: immunoblotting, 1:750, RIP: 4 µg) from Proteintech; RBMX (ab190352: immunoblotting, 1:1000) from Abcam; p‐STAT3 (ab76315: immunoblotting, 1:2000) from Abcam; FAP (sc‐65398: immunofluorescence, 1:100) from Santa Cruz; *α*‐SMA (55135‐1‐ap: immunofluorescence, 1:50) from Proteintech; STAT3 (10253‐2‐ap: immunoblotting, 1:1000, ChIP: 4 µg) from Proteintech; CD63 (561925: flow cytometry, per test 20 µL, human) from BD bioscience; CD63 (143903: flow cytometry, per test 0.5 µg, mouse) from Biolegend; F‐actin (40735ES75: immunofluorescence, 1:100) from YEASEN.

The inhibitors were as follows: STAT3 inhibitors (HY‐15146: 30 × 10^−6^ m for the in vitro assay) from MedChem Express; GW4869 (HY‐19363: 20 × 10^−6^ m for the in vitro assay) from MedChem Express; JAK inhibitor baricitinib (HY‐15315: 50 × 10^−6^ m for the in vitro assay) from MedChem Express.

##### Cell Lines and Culture

The cell lines T47D and HEK‐293T were obtained from the Type Culture Collection of the Chinese Academy of Sciences (Shanghai, China). These cell lines were authenticated by the analysis of short tandem repeat (STR) profiles and 100% matched the standard cell lines in the DSMZ data bank. These cells tested negative for cross‐contamination of other human cells and mycoplasma contamination. For 4‐hydroxytamoxifen treatment, tumor cells were cultured in phenol red‐free DMEM supplemented with 10% charcoal‐filtered FBS.

##### Patients and Samples

A total of 38 breast cancer tissue samples were obtained from the Department of General Surgery, Tangdu Hospital, Fourth Military Medical University (FMMU, Shaanxi, China) after receiving ethical approval and informed consent from the patients. Clinical staging of the breast cancer samples was performed according to the American Joint Committee on Breast Cancer Staging and Classification criteria (Table S3, Supporting Information). The study protocol was approved by the Ethics Committee of FMMU. Fresh breast cancer samples were washed with PBS and divided into two parts: the first was used for the isolation of BCs or CAFs, and the other was fixed with 10% formalin and embedded in paraffin for immunohistochemistry or immunofluorescence staining.

##### Clinical Specimens and Immunohistochemistry

Immunohistochemistry was performed as previously described.^[^ ^56^ ^]^ Briefly, sections (4 µm thick) of paraffin‐embedded samples were deparaffinized and rehydrated in a graded series of ethanol. After inactivation of endogenous peroxidase activity with 3% H_2_O_2_ in methanol for 10 min, the sections were washed three times in PBS and blocked with goat serum for 20 min. Then, they were incubated with primary antibodies in a humid container at 4 °C overnight. After the addition of PowerVision complex, tumor sections were incubated at 37 °C for 20 min followed by DAB labeling to develop a brown color. PBS was used in place of the primary antibody as a negative control. Staining for ER*α* was quantified using the immunohistochemistry H‐score as follows: H‐score = ∑Pi × (*i* + 1), where *i* is the intensity score (range 0–4) and Pi is the percentage of stained tumor cells at each intensity (range 0–100%). With respect to = ER*α* expression, each specimen was classified as “high” (H‐score>50) or “low” (H‐score<50).

##### Single‐Cell Sequencing

Isolation of single cells: All animal experiments were performed in accordance with a protocal approved by the Institutional Animal Care and Use Committee of FMMU. Primary breast cancer tissues were collected from MMTV‐PyMT mice (W6, W8, W10, and W12). The time from breast tumor sample collection to processing was within 30 min. Single‐cell suspensions of breast cancer tissues were obtained by mechanical dissociation and enzymatic digestion, and the resulting cell suspensions were filtered through a 40 µm nylon cell strainer. Dead cells were removed with a Dead Cell Removal Kit (Miltenyi Biotec), and the cell survival rate was generally above 99%. A total of 50 000 cells were loaded onto an individual 10–17 mm integrated fluidic circuit mRNA sequencing chip in a C1 Single‐Cell Auto Prep System (Fluidigm), and the loaded chips were microscopically examined to verify single‐cell loading.

cDNA Amplification: For cell lysis and cDNA synthesis and amplification, a SMARTer Ultra Low RNA Kit (Clontech) was used following the manufacturer's instructions. RNA spike‐ins 1, 4, and 7 from ArrayControl RNA Spikes (Thermo Fisher) were added to the lysis mix. The quantity and quality of the amplified cDNAs were measured using a Qubit 2.0 Fluorometer (Life Technologies) and 2100 Bioanalyzer (Agilent Technologies), respectively.

Droplet‐based scRNA‐seq. scRNA‐seq was performed at Genergy Bio (Shanghai, China). In total, 3000 single‐cell cDNAs were subjected to RNA sequencing. Briefly, single‐cell suspensions were converted to barcoded scRNA‐seq libraries by using a Chromium Single Cell 3′ Reagent Kit v2 (10× Genomics). Libraries were sequenced on an Illumina HiSeq4000. Data were analyzed and mapped to the mouse genome (mm10) using CellRanger software (10× Genomics).

##### ChIP Assay

Two primer sets were designed to flank putative STAT3 binding sites in the promoter region of CD63 or miR‐22. Details of the primer sequences are listed in Table S4 in the Supporting Information. Briefly, CD63^+^ CAFs or CD63^+^‐depleted CAFs were fixed with 1% paraformaldehyde and sonicated for 10 s each using a sonicator with a microtip in a 1.5 mL tube. Anti‐STAT3 antibody was applied to pull down chromatin associated with STAT3, and the chromatin–antibody complexes were collected with Protein G Agarose. After the complexes were washed and eluted from the beads, the crosslinkages were reversed at 65 °C overnight. The amounts of pulled‐down DNA fragments were then quantified by real‐time PCR and normalized against the genomic DNA preparation from the same cells. Each group was assessed in triplicate.

##### Statistical Analysis

The data are presented as the means ± S.E.M. from at least three independent experiments. Statistical analysis was performed using GraphPad Prism 8.3.0 software. A random number table was used to randomize the mice into control and treatment groups, and the numbers of mice used were determined on the basis of the pretests and previous experience with similar experiments. The statistical tests were two‐sided, and a value of *p* < 0.05 was considered statistically significant.

## Conflict of Interest

The authors declare no conflict of interest.

## Author Contributions

Y.G. and X.L. contributed equally to this work. C.Z., W.Z., M.L., and Y.Z. contributed in project conceptualization and supervision; C.Z., W.Z., M.L., Y.G., X.L., C. Z., C.L., Q.H., and W.L. contributed in investigation; C.Z., W.Z., M.L., Y.G., X.L., C.Z., and W.Z contributed in data curation; C.Z., W.Z., M.L., Y.G., X.L., C.Z., and C.L. contributed in methodology and formal analysis and visualization; C.Z., Y.G., and X.L. contributed in writing the original draft; C.Z. and Y.G. contributed in writing, reviewing, and editing; D.F. and H.Z. contributed in providing resources (clinical samples); C.Z., Y.Z., Y.G., K.Z., and S.W. contributed in funding acquisition.

## Supporting information

Supporting Information
