# Supplementary material for: CD63+ Cancer‐Associated Fibroblasts Confer Tamoxifen Resistance to Breast Cancer Cells through Exosomal miR‐22
Source: Adv Sci (Weinh). 2020 Sep 24;7(21):2002518. doi: 10.1002/advs.202002518 (PMC7610308; doi:10.1002/advs.202002518)
Supplement: Supplementary file 1 — Supporting Information [file ADVS-7-2002518-s001.pdf]

© 2020 Wiley-VCH GmbH

ADVANCED SCIENCE
Open Access

Supporting Information

for *Adv. Sci.,* DOI: 10.1002/advs.202002518

CD63$^+$ Cancer-Associated Fibroblasts Confer Tamoxifen Resistance to Breast Cancer Cells through Exosomal miR-22

Yuan Gao, Xiaoju Li, Cheng Zeng, Chenlin Liu, Qiang Hao, Weina Li, Kuo Zhang, Wangqian Zhang, Shuning Wang, Huadong Zhao, Dong Fan, Meng Li, Yingqi Zhang, Wei Zhang,* and Cun Zhang**

CD63+ Cancer-Associated Fibroblasts Confer Tamoxifen Resistance to Breast Cancer Cells through Exosomal miR-22

Yuan Gao[1#], Xiaoju Li[1#], Cheng Zeng[1,3], Chenlin Liu[1], Qiang Hao[1], Weina Li[1], Kuo Zhang[1], Wangqian Zhang[1], Shuning Wang[1], Huadong Zhao[2], Dong Fan[2], Meng Li [1], Yingqi Zhang[1*], Wei Zhang[1*], Cun Zhang[1*]

1. The State Key Laboratory of Cancer Biology, Biotechnology Center, School of Pharmacy, The Fourth Military Medical University, 710032 Xi'an, People's Republic of China
2. Department of General Surgery, Tangdu Hospital, The Fourth Military Medical University, 710038 Xi'an, People's Republic of China
3. Institute of Material Medical, School of Pharmacy, The Fourth Military Medical University, Xi'an 710032, People's Republic of China

---

[#]These authors contributed equally to this article.

[*]Corresponding authors: Cun Zhang, Ph.D., zhangcun@fmmu.edu.cn; Phone: 86-29-84774774; Wei Zhang, Ph.D., zhangw90@fmmu.edu.cn; Phone: 86-29-84711511; Yingqi Zhang, Ph.D., zhangyqh@fmmu.edu.cn; Phone: 86-29-84774773; Address: The State Key Laboratory of Cancer Biology, The Fourth Military Medical University, 169 Changle West Road, 710032, Xi'an, P. R. China

Supplementary Figure

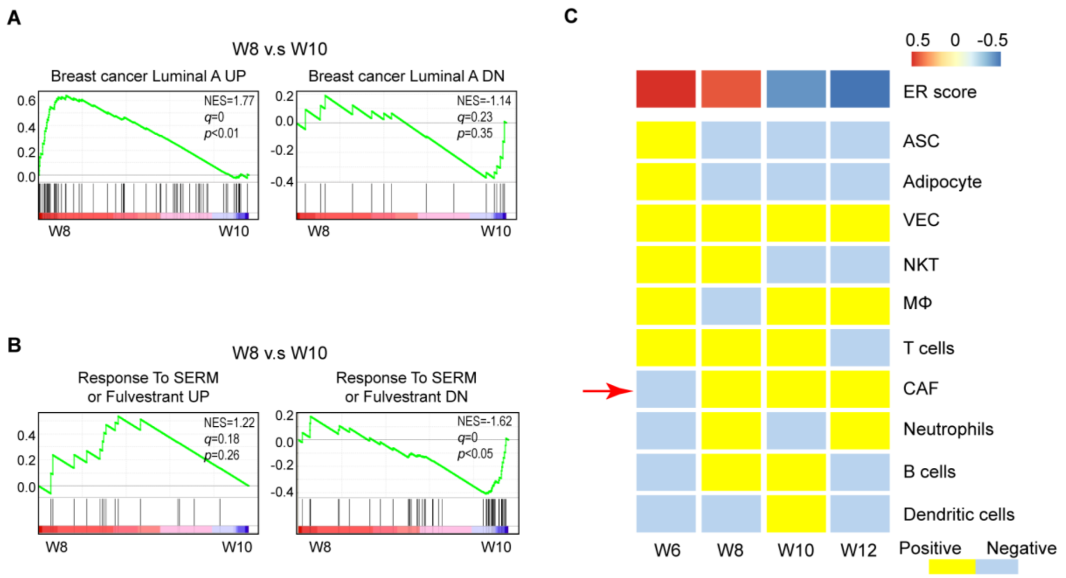

Figure S1. Single-cell sequencing reveals that CAFs are associated with a poor tamoxifen response in breast cancer. Related to Figure 2. (A) GSEA revealed the enrichment of gene sets related to "Luminal A" in the ranked gene list of all BCs from W8 MMTV-PyMT mice versus all BCs from W10 MMTV-PyMT mice. **(B)** GSEA revealed the enrichment of gene sets related to "Response to tamoxifen or fulvestrant" in the ranked gene list of all BCs from W8 MMTV-PyMT mice versus all BCs from W10 MMTV-PyMT mice **(C)** Through scRNA-seq, we identified 10 major cell types in the TME. Then, these cell types in the TME of each group are shown in the heatmap. Positive means that this cell type does exist in this group. The corresponding ERα H-scores (related to Figure 1E) of each group are also shown.

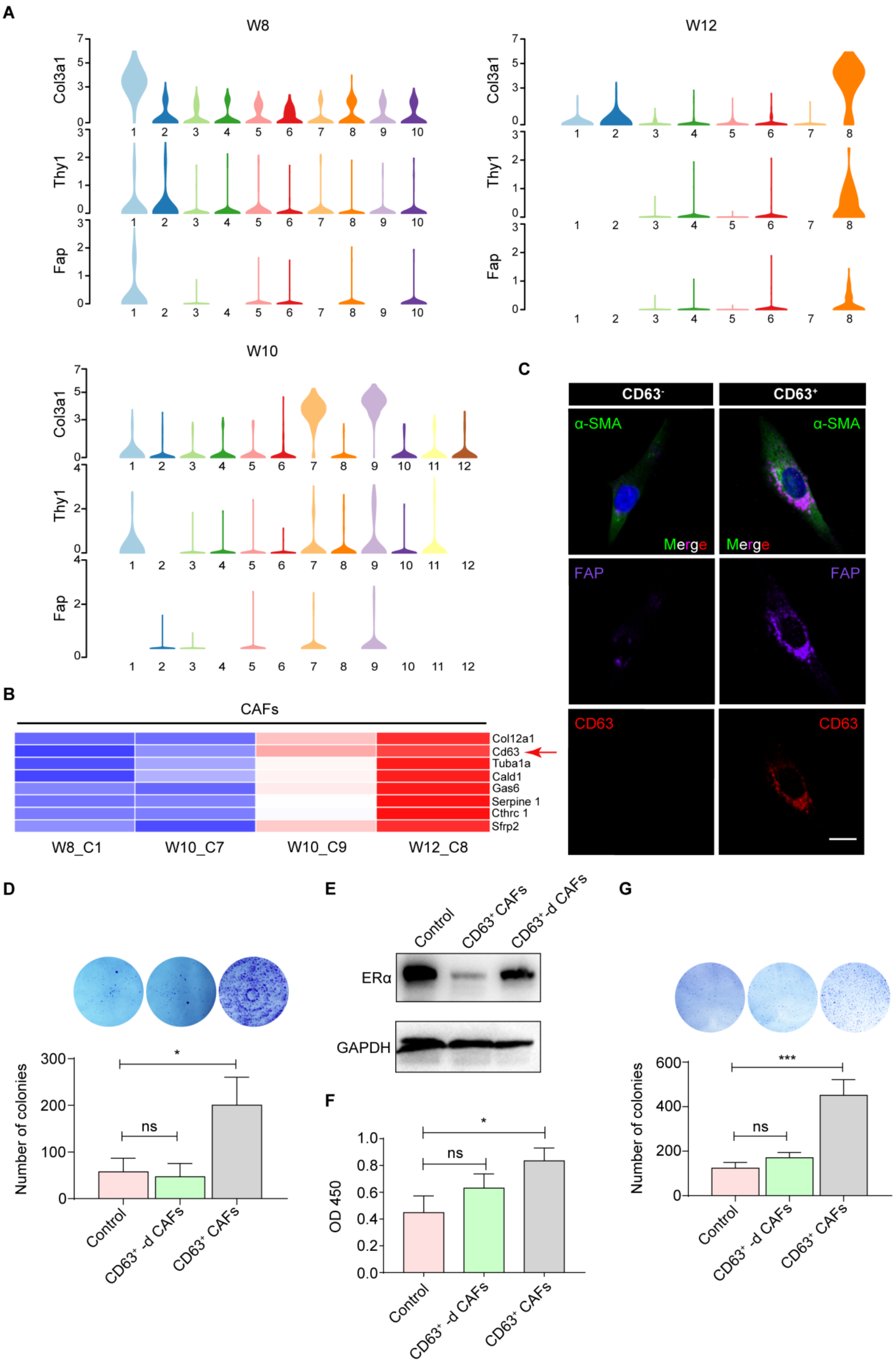

Figure S2. CD63$^+$ CAFs induce ERα downregulation and tamoxifen resistance. Related to Figure 3. (A) Violin plots showing the expression distribution of Col3a1,

Thy1 and FAP in each cluster. **(B)** Heatmap representing DEGs of the CAFs. The red arrow indicates CD63 expression. Cluster 9 of W10 (CAFs) and cluster 8 of W12 (CAFs) showed high CD63 expression. **(C)** Representative images of α-SMA, FAP, and CD63 immunofluorescent staining in $CD63^+$ CAFs and $CD63^-$ CAFs (scale bar, 10 μm). **(D)** Colony formation and counts of T47D cells. T47D cells alone or cocultured with $CD63^+$ CAFs or $CD63^+$ -depleted CAFs in the presence of 4-hydroxytamoxifen. **(E)** Western blotting was conducted to detect ERα expression in ERα-positive BCs derived from W8 MMTV-PyMT mice (or cocultured with $CD63^+$ CAFs or $CD63^+$ -depleted CAFs). **(F)** Viability of ERα-positive BCs either derived from W8 MMTV-PyMT mice alone or cocultured with $CD63^+$ CAFs or $CD63^+$ -depleted CAFs in the presence of 4-hydroxytamoxifen. **(G)** Colony formation and counts of ERα-positive BCs derived from W8 MMTV-PyMT mice. BCs alone or cocultured with $CD63^+$ CAFs or $CD63^+$ -depleted CAFs in the presence of 4-hydroxytamoxifen. (**D, F, G)** The data are shown as the means±s.e.m. ns $P>0.05$, *$P<0.05$. ANOVA with Dunnett's *t*-test.

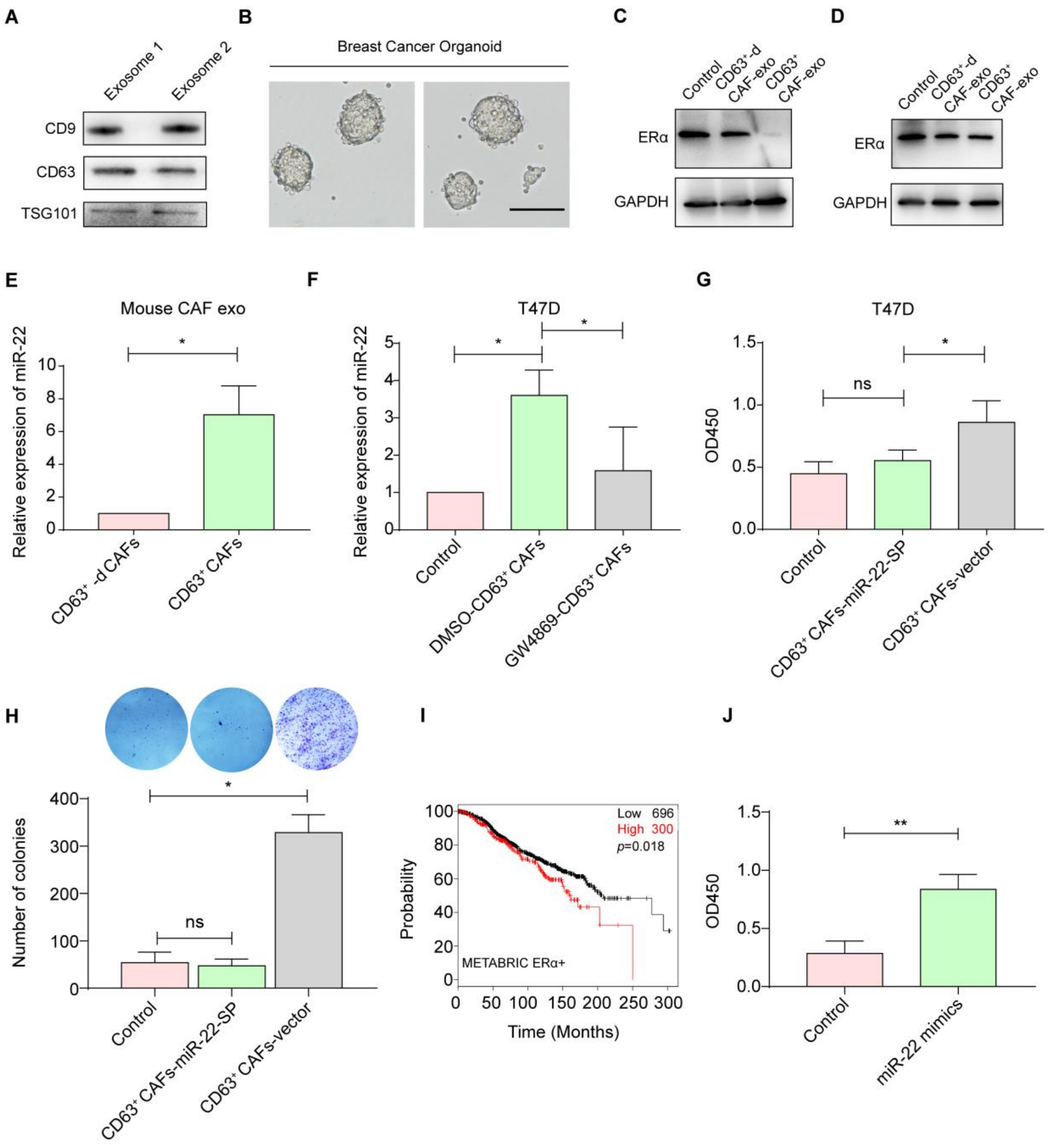

Figure S3. CD63$^+$ CAF-derived exosomal miR-22 promotes tamoxifen resistance. Related to Figure 4. (A) Western blotting analysis of indicated proteins in exosomes from CAFs. **(B)** Representative images of human breast cancer organoids derived from ERα-positive patients. Scale bar, 100 μm. **(C)** ERα expression in ERα-positive BCs derived from W8 MMTV-PyMT mice treated with vehicle, CD63$^+$ CAFs or CD63$^+$ -depleted CAF-derived exosomes. **(D)** ERα expression in ERα-positive BCs derived from W8 MMTV-PyMT mice treated with vehicle, Dicer knockdown CD63$^+$ CAFs or Dicer knockdown CD63$^+$ -depleted CAF-derived exosomes. **(E)** miR-22

expression in mouse $CD63^+$ CAFs and $CD63^+$ -depleted CAF-derived exosomes was determined using real-time PCR. **(F)** T47D cells were either cultured alone or cocultured with DMSO-treated $CD63^+$ CAFs or GW4869-treated $CD63^+$ CAFs for 24 h. miR-22 expression was then detected in T47D cells using real-time PCR. GW4869 could block the secretion of exosomes. **(G)** Viability of T47D cells in the presence of 4-hydroxytamoxifen. Control, treated with vehicle; $CD63^+$ CAF-miR-22-sp, treated with exosomes derived from $CD63^+$ CAFs with downregulated miR-22 expression; $CD63^+$ CAF-vector, treated with exosomes derived from control $CD63^+$ CAFs. (**H**) Colony formation and counts of T47D cells in the presence of 4-hydroxytamoxifen. Control, treated with vehicle; $CD63^+$ CAF-miR-22-sp, treated with exosomes derived from $CD63^+$ CAFs with downregulated miR-22 expression; $CD63^+$ CAF-vector, treated with exosomes derived from control $CD63^+$ CAFs. (**I**) Overall survival (months) analysis of miR-22 in ERα-positive breast cancer patients. Data from METABRIC. (**J**) Viability of T47D cells in the presence of 4-hydroxytamoxifen. Control, transfected with control mimics; miR-22 mimics, transfected with miR-22 mimics. The data are shown as the means±s.e.m. n.s., $P>0.05$. $*P<0.05$. (**E)** Paired *t*-test. (**F-H)** ANOVA with Dunnett's *t*-test. (**I)** Log-rank test. (**J)** Unpaired *t*-test.

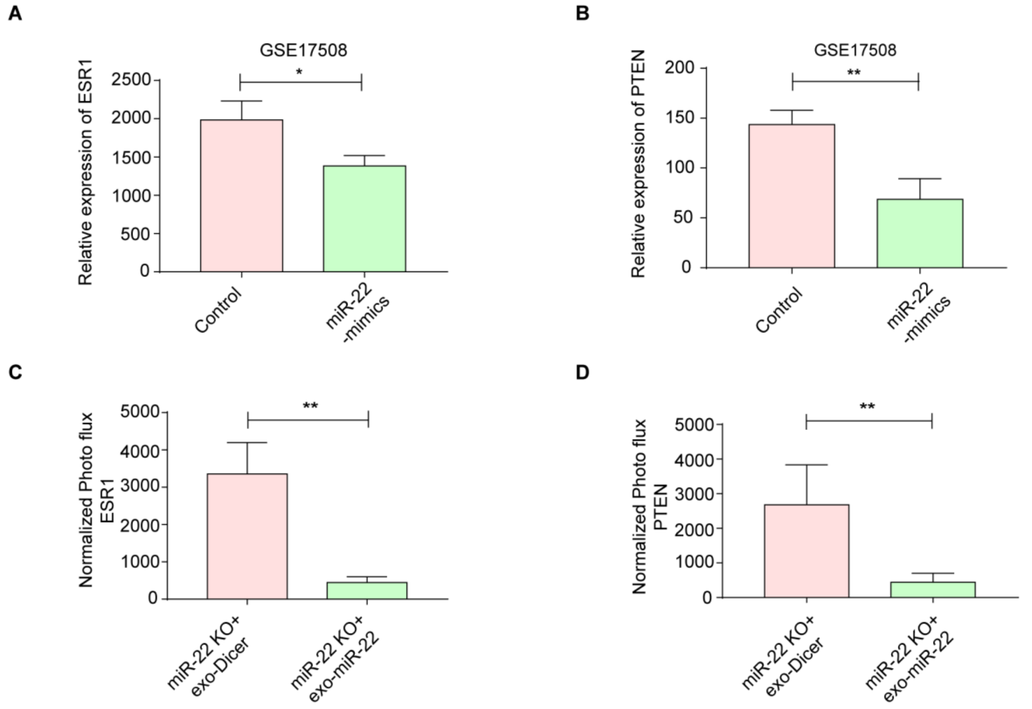

Figure S4. ESR1 and PTEN are direct targets of exosomal miR-22 in breast cancer cells. Related to Figure 5. (A) The mRNA expression of ERα in MCF-7 cells transfected with miR-22 mimics or control mimics (data from GSE17508). **(B)** The mRNA expression of PTEN in MCF-7 cells transfected with miR-22 mimics or control mimics (data from GSE17508). **(C)** Bioluminescence counts in the breasts of mice indicating *in vivo* regulation of ERα expression. **(D)** Bioluminescence counts in the breasts of mice indicating *in vivo* regulation of PTEN expression. The data are shown as the means±s.e.m. * $P<0.05$. *$P<0.05$. (A-**D)** Unpaired *t*-test.

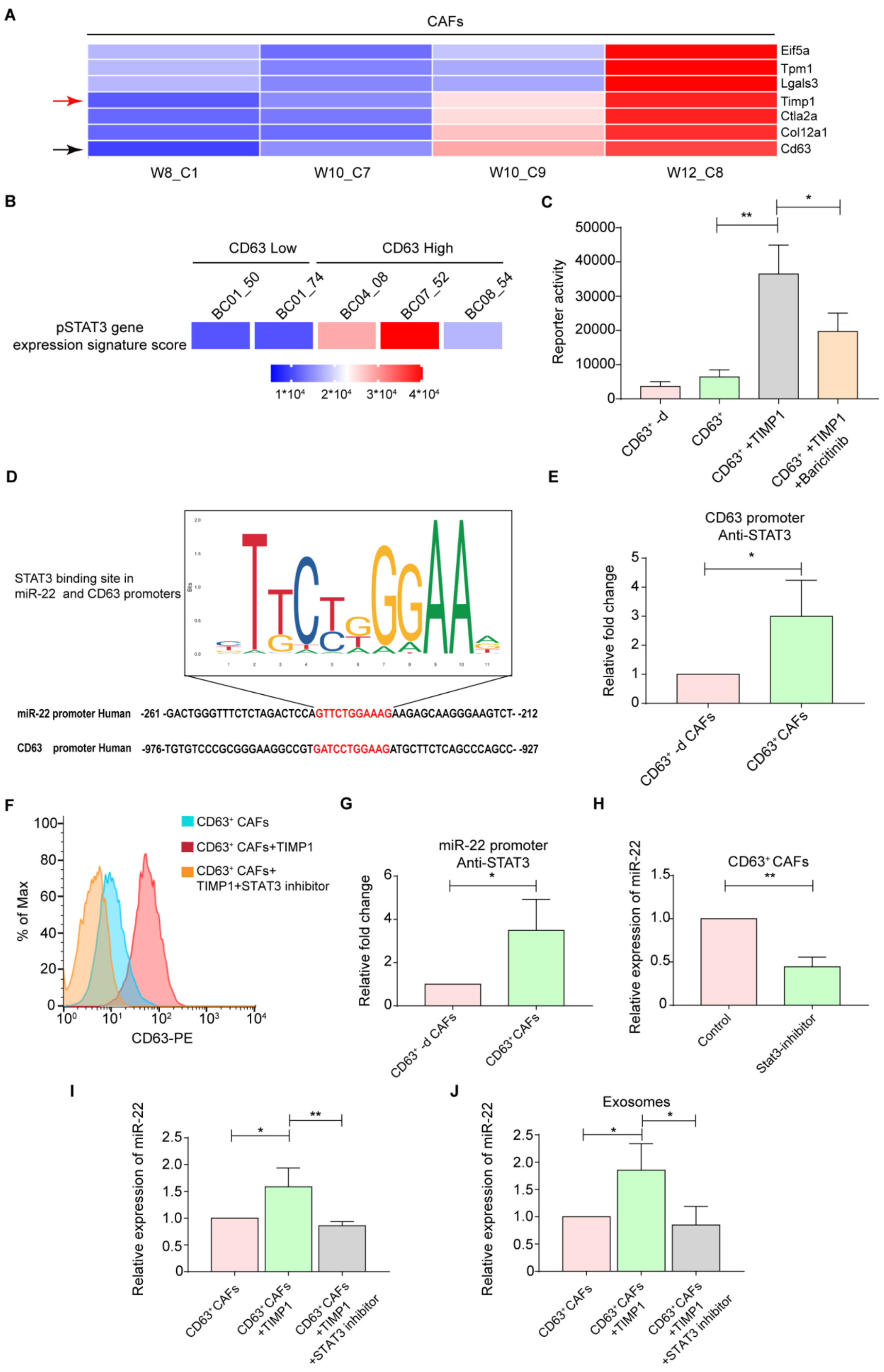

Figure S5. Prolonged STAT3 activation maintains the phenotypes and functions of CD63$^+$ CAFs. Related to Figure 6. (A) Heatmap representing DEGs of the CAFs.

The black arrow indicates CD63 expression, and the red arrow indicates TIMP1 expression. Cluster 9 of W10 (CAFs) and cluster 8 of W12 (CAFs), which have high CD63 expression, also showed high TIMP1 expression. **(B)** Heatmap representing pSTAT3 gene expression signature scores of CAFs with high or low CD63 expression. The human primary breast cancer scRNA-seq data were from GSE75688**. (C)** Luciferase signals of CAFs with stable transduction of STAT3 reporter plasmids were quantified. $CD63^+$ -depleted CAFs, $CD63^+$ CAFs in the presence of blocking antibodies against TIMP1, and $CD63^+$ CAFs in the presence of blocking antibodies against TIMP1 and either 5 ng/ml human recombinant TIMP1 or the JAK inhibitor baricitinib were assessed. **(D)** STAT3-binding elements on the promoters of human miR-22 and CD63 genes were predicted by JASPAR. **(E)** The amounts of DNA fragments from the CD63 promoter were normalized to the total input DNA from $CD63^+$ CAFs or $CD63^+$ -depleted CAFs precipitated by the anti-STAT3 monoclonal antibody. The amplification regions of the ChIP primers contain the potential binding site shown in D. **(F)** Flow cytometric analysis of CD63 expression in different CAFs. $CD63^+$ CAFs: $CD63^+$ CAFs in the presence of blocking antibodies against TIMP1, $CD63^+$ CAFs+TIMP1: $CD63^+$ CAFs treated with blocking antibodies against TIMP1 followed by treatment with 5 ng/ml human recombinant TIMP1, $CD63^+$ CAFs+TIMP1+STAT3 inhibitor: $CD63^+$ CAFs treated with blocking antibodies against TIMP1 followed by treatment with 5 ng/ml human recombinant TIMP1 and a STAT3 inhibitor. **(G)** The amounts of DNA fragments from the miR-22 promoter that were normalized to the total input DNA from $CD63^+$ CAFs or $CD63^+$ -depleted CAFs

precipitated by anti-STAT3 monoclonal antibody. The amplification regions of ChIP primers contain the potential binding site shown in D. **(H)** The miR-22 expression level in control $CD63^+$ CAFs or $CD63^+$ CAFs treated with the STAT3 inhibitor was determined using real-time PCR. **(I-J)** The miR-22 expression level was determined using real-time PCR. $CD63^+$ CAFs: $CD63^+$ CAFs in the presence of blocking antibodies against TIMP1, $CD63^+$ CAFs+TIMP1: $CD63^+$ CAFs treated with blocking antibodies against TIMP1 followed by treatment with 5 ng/ml human recombinant TIMP1, $CD63^+$ CAFs+TIMP1+STAT3 inhibitor: $CD63^+$ CAFs treated with blocking antibodies against TIMP1 followed by treatment with 5 ng/ml human recombinant TIMP1 and a STAT3 inhibitor. (I) Data from different $CD63^+$ CAFs. (J) Data from the exosomes of different $CD63^+$ CAFs. **(C, E, G-J)** The data are shown as the means±s.e.m. $*P<0.05$. $**P<0.01$. **(C, I-J)** ANOVA with Dunnett's *t*-test. **(E, G-H)** Paired *t*-test.

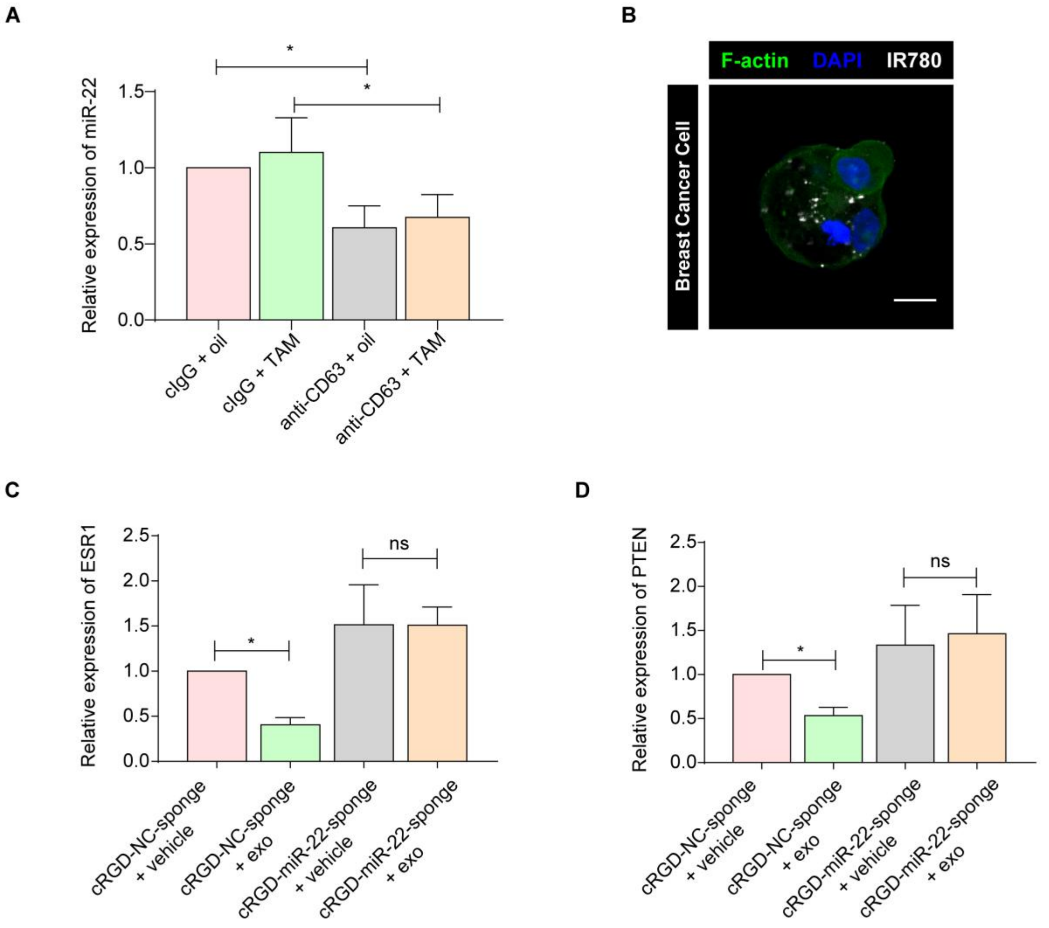

Figure S6. Pharmacological inhibition of CD63$^+$ CAF activity sensitizes breast tumors to tamoxifen therapy. Related to Figure 8. (A) ERα-positive breast cancer cells mixed with CD63$^+$ CAFs were implanted into nude mice, which were then coadministered tamoxifen and anti-CD63 neutralizing antibody. Corn oil and a nonspecific IgG1 isotype control antibody were used as the respective negative controls. The plasma exosomal miR-22 level of each group was detected using real-time PCR. (**B**) Representative confocal images of BCs incubated with IR780-labeled cRGD-decorated nanoparticles for 2 h. IR780 (white) and F-actin (green), scale bar, 10 μm. (**C-D**) The combination of CD63$^+$ CAF-derived exosomes and cRGD-miR-22-sponge nanoparticles was administered to BCs, with PBS and

cRGD-NC-sponge nanoparticles serving as the respective negative controls. (C) The expression of ESR1 in BCs was determined using real-time PCR. (D) The expression of PTEN in BCs was determined using real-time PCR. **(A, C-D)** The data are shown as the means±s.e.m. *P<0.05. ns P>0.05. **(A, C-D)** ANOVA with Tukey's *t*-test.

Supplementary table

Table S1: A list of differentially expressed genes that define the clusters

Group	Cluster	Cell	Key Marker gene
W6	Cluster 1	BC01	Epcam
	Cluster 2	ASC	Pi16 Plac8
	Cluster 3	Adipocyte	FABP4 Car3 Plin3 Adipoq
	Cluster 4	VEC	PECAM1 Hbb-bs CDH5
	Cluster 5	BC02	Epcam
	Cluster 6	Mφ	Cybb Emp1 Msr1
	Cluster 7	T cells	CD3d CD3e CD3g CD27 CD28
	Cluster 8	VEC	Mef2c Hbb-bt CDH5
	Cluster 9	NKT	AW112010 CCL5 CD3 CD44 CD4 CD8
	Cluster 10	ASC	sfrp2 Col14a1 FSTL1
	Cluster 11	Mφ	Cybb Emp1 Msr1
W8	Cluster 1	CAF	Col1A1 Col3A1 THY1 FAP
	Cluster 2	T cells	CD3d CD3e CD3g CD27 CD28
	Cluster 3	BC04	EPCAM
	Cluster 4	B cells	CD19 CD79a CD79b
	Cluster 5	B cells	CD19 CD79a CD79b
	Cluster 6	BC03	Epcam
	Cluster 7	VEC	Vwf Hba-a1 Hbb-bs Hbb-bt
	Cluster 8	Neutrophils	Csf3r DYSF BST1
	Cluster 9	Neutrophils	Csf3r FCGR3 HPSE
	Cluster 10	NKT	FTL1 H2-aa AW112010 CCL5
W10	Cluster 1	T cells	CD3d CD3e CD3g CD27 CD28
	Cluster 2	BC05	Epcam
	Cluster 3	Mφ	Cybb Emp1 Msr1
	Cluster 4	B cells	CD19 CD79a CD79b
	Cluster 5	BC06	Epcam
	Cluster 6	BC07	Epcam
	Cluster 7	CAF	Col1A1 Col3A1 THY1 FAP
	Cluster 8	B cells	CD19 CD79a CD79b
	Cluster 9	CAF	Col1A1 Col3A1 THY1 FAP
	Cluster 10	Dendritic cells	CCL17 CCL22
	Cluster 11	B cells	CD19 CD79b abcb4
	Cluster 12	VEC	CDH5 CD36 TCF4
W12	Cluster 1	BC08	Epcam
	Cluster 2	VEC	mt-nd4l HBB-BS HBB-Bt
	Cluster 3	BC09	Epcam

Cluster 4	BC10	Epcam
Cluster 5	BC11	Epcam
Cluster 6	Mφ	Cybb Emp1 Msr1
Cluster 7	Neutrophils	Cxcr2 Gos2 Csf3r
Cluster 8	CAF	Col1A1 Col3A1 THY1 FAP

Table S2: The miRNA expression levels in exosomes derived from $CD63^+$ CAFs and $CD63^+$ -depleted CAFs

miRNA_ID	$CD63^+$-d CAFs	$CD63^+$ CAFs	Fold Change
hsa-miR-20a-5p	2326	44119	18.9677558
hsa-miR-17-5p	1295	22260	17.18918919
hsa-miR-93-5p	1708	23304	13.6440281
hsa-miR-103a-3p	4182	38566	9.221903396
hsa-miR-190b	1473	12958	8.797012899
hsa-miR-7-5p	1259	9368	7.440826052
hsa-miR-203a-3p	4098	29023	7.082235237
hsa-miR-152-3p	7264	49779	6.852835903
hsa-miR-429	3040	19585	6.442434211
hsa-miR-589-5p	1218	7804	6.407224959
hsa-miR-200b-3p	16383	97637	5.959653299
hsa-miR-140-3p	4057	24109	5.9425684
hsa-miR-30a-5p	3502	20188	5.764705882
hsa-miR-148b-3p	12361	70565	5.708680527
hsa-miR-191-5p	19862	112382	5.658141174
hsa-miR-199a-3p	3180	17811	5.600943396
hsa-miR-199b-3p	3180	17811	5.600943396
hsa-miR-16-5p	1132	6328	5.590106007
hsa-miR-340-5p	1226	6819	5.561990212
hsa-miR-186-5p	2566	13351	5.203039751
hsa-miR-23a-3p	3849	19075	4.955832684
hsa-miR-101-3p	16855	81808	4.853633937
hsa-miR-493-5p	3311	15359	4.638779825
hsa-miR-142-5p	1108	5046	4.554151625
hsa-miR-30c-5p	2759	12518	4.537151142
hsa-miR-125b-5p	3315	15004	4.526093514
hsa-let-7g-5p	23786	106398	4.473135458
hsa-miR-330-3p	1282	5690	4.438377535
hsa-miR-28-3p	1809	7287	4.028192371
hsa-miR-26b-5p	6574	26297	4.000152114

hsa-miR-532-5p	15757	62975	3.996636416
hsa-miR-106b-3p	5817	22391	3.849235001
hsa-miR-92a-3p	36437	139799	3.836731893
hsa-miR-30e-3p	1071	4034	3.766573296
hsa-miR-200a-3p	1131	4256	3.763041556
hsa-miR-433-3p	1193	4418	3.70326907
hsa-miR-92b-3p	1959	7012	3.579377233
hsa-miR-194-5p	1137	4012	3.528583993
hsa-miR-25-3p	34171	119502	3.497175968
hsa-miR-30a-3p	1408	4912	3.488636364
hsa-miR-484	1482	5105	3.444669366
hsa-miR-30d-5p	17712	60500	3.415763324
hsa-miR-192-5p	14971	50710	3.387215283
hsa-miR-181b-5p	2350	7934	3.376170213
hsa-miR-100-5p	27443	92618	3.374922567
hsa-miR-200c-3p	4403	13904	3.157846923
hsa-miR-99b-3p	2711	8540	3.150129104
hsa-miR-21-5p	47456	144159	3.037740223
hsa-miR-182-5p	2409	7128	2.95890411
hsa-miR-629-5p	1945	5674	2.91722365
hsa-miR-423-3p	24835	72215	2.907791423
hsa-miR-224-5p	1268	3592	2.832807571
hsa-miR-10b-5p	27666	76759	2.774488542
hsa-miR-16-2-3p	1676	4605	2.747613365
hsa-miR-142-3p	1104	3017	2.732789855
hsa-miR-139-5p	1615	4215	2.609907121
hsa-let-7i-5p	29163	75199	2.578575592
hsa-miR-27b-3p	31620	81058	2.563504111
hsa-miR-196a-5p	13498	34448	2.55208179
hsa-miR-10a-5p	11917	29886	2.507845934
hsa-miR-125a-5p	4379	10787	2.463347796
hsa-miR-22-3p	2678	6553	2.446975355
hsa-miR-378a-3p	17614	42662	2.422050642
hsa-miR-127-3p	18953	45788	2.415870838
hsa-miR-411-5p	3351	8040	2.399283796
hsa-miR-24-3p	21327	50854	2.384489145
hsa-miR-26a-5p	59595	139269	2.336924239
hsa-miR-148a-3p	790310	1827348	2.312191419
hsa-miR-382-5p	2877	6466	2.247480014
hsa-miR-183-5p	8718	19561	2.243748566
hsa-miR-375	7015	15671	2.233927299
hsa-miR-1468-5p	5600	12410	2.216071429
hsa-miR-1180-3p	3719	8176	2.198440441
hsa-miR-379-5p	10733	23191	2.160719277

hsa-miR-143-3p	52159	111876	2.144903085
hsa-let-7f-5p	77094	163573	2.121734506
hsa-miR-215-5p	2345	4975	2.121535181
hsa-miR-1307-3p	6016	12375	2.057014628
hsa-miR-7706	1893	3878	2.048600106
hsa-miR-381-3p	5364	10865	2.025540641
hsa-miR-328-3p	4968	9977	2.008252818

Table S3: Breast carcinoma characteristics

Variables	Number	
**Age(year)**		
<50	22	57.9%
>50	16	42.1%
Total	38	
**Clinical**		
AJCC Ⅰ	9	23.7%
AJCC Ⅱ	15	39.5%
AJCC Ⅲ	14	36.8%
Total	38	
**ER status**		
ER+	32	84.2%
ER-	6	15.8%
Total	38	
**PR status**		
PR+	23	60.5%
PR-	15	39.5%
Toal	38	
**HER2 status**		
HER2+	10	26.3%
HER2-	28	73.7%
Total	38	

Table S4: The sequence of primer sets for ChIP

ID	Forward	Reverse
CD63	CCGCTCCTCCTCCACAGCC	CACTCCTTGGGCTCTTAAGGTCCTG
MIR-22	AGAGCAGGACTGGGTTTCTCTAGAC	ACCCACTCGCTGGCCATCAG

Table S5: The sequence of primers

ID	F（5'-3'）	R（5'-3'）
MMTV-PyMT	GGAAGCAAGTACTTCACAAGGG	GGAAAGTCACTAGGAGCAGGG
Mir22#1	GGGTTCTACACCCTGCCCTTTG	TAATCTTGGGGAGGTGGAGTCAC
Mir22#2	GGGTTCTACACCCTGCCCTTTG	CTTGCCACTGAAGAACTACTGCGG

Table S6: The sequence of siRNAs

ID	sense（5'-3'）	antisense（5'-3'）
SRSF1#1	ACAUAAGAUGAUUGGUGACTT	GUCACCAAUCAUCUUAUGUTT
SRSF1#2	GCAUCUACGUGGGUAACUUTT	AAGUUACCCACGUAGAUGCTT
RBMX#1	GCCAGAGACAUGAAUGGAATT	UUCCAUUCAUGUCUCUGGCTT
RBMX#2	GGUCGUGAUCGUGACUAUUTT	AAUAGUCACGAUCACGACCTT
Negative control	UUCUCCGAACGUGUCACGUTT	ACGUGACACGUUCGGAGAATT

Supplementary Method

Exosome purification

For exosome isolation from CAFs, RPMI 1640 medium with 10% exosome-depleted FBS (ultracentrifugation at 120,000 ×g for 16 h) was used. Exosomes were isolated by differential centrifugation. In detail, supernatant fractions collected from cells cultured for 48-72 h were centrifuged at 800 ×g for 5 min, 2000 ×g for 10 min and 10000 ×g for 30 min. The supernatant was then filtered through 0.22 μm filters and subjected to a final centrifugation at 100,000 ×g for 2 h. The supernatant was aspirated, and the pellet was resuspended in PBS. The concentration of exosomes was determined by measuring total exosomal protein using a Pierce BCA Protein Assay Kit (Thermo Scientific, USA) according to the manufacturer's instructions. NanoSight and transmission electron microscopy were used to determine the size distribution, concentration and morphology of the exosomes.

Mice

All animal experiments were performed in accordance with a protocol approved by the Institutional Animal Care and Use Committee of FMMU. Mice were bred and maintained in a specific pathogen-free barrier facility. FVB/N-Tg(MMTV-PyMT)634 Mul/J transgenic mice were purchased from The Jackson Laboratory. miR-22 KO mice (C57BL/6N background) were purchased from the model animal research center of Nanjing University. DNA was prepared from either ear or tail biopsies following a common protocol for tissue lysis, nucleic acid extraction and purification. The primer

pairs used are listed in Table S5. Female mice were used in this study. Mice deficient for miR-22 were originally on the C57BL/6 background and were backcrossed to the FVB/N background for ten generations before experiments were performed. Furthermore, miR-22+/− mice were crossed with PyMT mice to produce two groups of female mice: MMTV-PyMT+Mir22−/− mice (miR-22 KO) and MMTV-PyMT+Mir22+/+ mice (WT). In all cases, WT littermate mice were used as controls for the KO mice.

Mouse experiments

Tamoxifen administration in the MMTV-PyMT mouse model. Tamoxifen (Sigma) was dissolved in corn oil by heating the mixture to 55 °C and i.p. administered at a dose of 2 mg/mouse every three days. Tumor size was measured every three days with a caliper, and the tumor volume was calculated as length × width^2 × (π/6).

Combined treatment of anti-CD63 antibody and tamoxifen. Breast cancer tissues were collected from ERα-positive breast cancer patients who underwent tumor resection at Tangdu Hospital, FMMU (Xi'an, China) after receiving ethical approval and informed consent from the patients. A Tumor Dissociation Kit (Miltenyi Biotec) was used to generate single-cell suspensions, and a Tumor Cell Isolation Kit (Miltenyi Biotec) was used to isolate BCs from breast cancer tissues. Purified breast cancer cells were cultured in DMEM supplemented with 20% FBS. A total of 3*10^6 breast cancer cells mixed with CD63$^+$ CAFs at a ratio of 1:3 were implanted into the fat pads of 6-week-old female nude mice under pathogen-free conditions. When the tumors

reached approximately 4 mm in diameter, tamoxifen (2 mg/mouse, i.p.) and anti-CD63 antibody (BioLegend, 353040, 2.5 mg/kg, i.p.) were administered every three days. A nonspecific IgG1 isotype control antibody (BioLegend, 400166, 2.5 mg/kg, i.p.) was used as a negative control. The tumor size was measured every week with a caliper, and the tumor volume was calculated as length × width^2 × (π/6). A week before tumor cell inoculation, each mouse was implanted with a 17β-estradiol pellet.

In vivo regulation of ERα and PTEN. Female miR-22 KO mice were orthotopically injected with adenovirus bearing the 3′UTR of ERα or PTEN fused to the luciferase gene. The next day, mice received an i.v. injection of exosomes isolated from $3*10^6$ $CD63^+$ CAFs with either Dicer knockdown or miR-22 overexpression. Twenty-four hours later, *in vivo* luminescence was measured on an IVIS imaging system after mice were administered D-Luciferin following the manufacturer's protocol.

Combined treatment of nanoparticles and tamoxifen. The cRGD-miR-22-sponge nanoparticles were constructed by Nanjing Nanoeast Biotech. In brief, 75 mg of lecithin, 25 mg of DSPE-PEG-COOH, 10 mg of cholesterol and 1 mg of IR780 were dissolved in 5 ml of chloroform. This solution was fully rotated-vaporized until a thin film formed. Then, 20 nmol miR-22 sponge or NC sponge dissolved in DEPC-treated water was added to the film at low temperature. Finally, cRGD was added dropwise (cRGD:DSPE-PEG-COOH=1:1). Excess products were removed using an Amicon Ultra-15 Centrifugal Filter Unit (Millipore, USA). Nine-week-old female MMTV-PyMT recipient mice were i.v. injected with 0.5 mg/kg cRGD-miR-22-sponge

nanoparticles every three days and i.p. injected with tamoxifen (2 mg/mouse) every three days. cRGD-NC-sponge nanoparticles and corn oil were used as the respective negative controls. Tumor size was measured every three days with a caliper, and tumor volume was calculated as length × width^2 × (π/6).

Cell transfection

miR-22 mimics or miR-22 sponges (RIBOBIO, China) were used to enhance or knockdown miR-22 expression, respectively. siRNA molecules (Gene Pharma, China) were used to knockdown RBMX or SFRS1 expression (Table S6). siRNAs or miRNA mimics were transfected into cells using Lipofectamine 3000 (Invitrogen, USA) following the manufacturer’s instructions.

Flow cytometry

For cell surface marker analysis, cells were digested into single cell suspension and washed twice with PBS. Cell suspensions were incubated with mouse or human Fc Block (BD Biosciences), and then stained for surface markers.Washing by PBS for two times. Then the expression of surface markers was analyzed with a flow cytometer (BD FACSVerse) and FlowJo software.

qRT–PCR

Total RNA was isolated from cultured cells or exosomes with RNAiso Plus (Takara, Dalian, China). For, miRNA quantification, cDNA was synthesized with the

miDETECT A Track^{TM} miRNA qRT-PCR Stater Kit (RIBOBIO, China). Then, cDNA and ChamQ^{TM} SYBR$^{®}$ qPCR Master Mix (Vazyme, China) were used for real-time PCR in a Prism 7500 real-time thermocycler (Applied Biosystems, USA) according to the manufacturer's instructions. The results were analyzed by the relative quantitation $2^{-\Delta\Delta CT}$ and U6 was used as internal control. The primers for U6, miR-22, miR-152-3p and miR-148a were designed by RiboBio (Guangzhou, China) using the Bulge-loopTM miRNA qRT-PCR Primer sets (a uni-RT primer and a uni-reverse primer in the kit; one specifically designed miRNA forward primer for qPCR for each set). Each group was analyzed in triplicate.

Western blot analysis

The cells were lysed on ice, according to the instructions. Protease Inhibitor Cocktail (MedChem Express, USA) was used in cell lysates to increase protein stability. After SDS-PAGE, the proteins were transferred to PVDF membranes (0.22 μm, Invitrogen), using a Bio-Rad Semi-Dry Electrophoretic Transfer Cell. Western blot analyzes were performed, using corresponding specific antibodies, followed by HRP conjugated IgG antibody. An enhanced chemiluminescence against HRP was used for the visualization of immunoreactive proteins.

Immunofluorescence staining

For breast cancer specimens staining, paraffin-embedded samples were sectioned at 4 μm thickness. Then, sections were blocked with goat serum at room temperature for

20 min. Cells for immunofluorescence were seeded on glass plates at 37°C overnight. The cells were then washed twice with cold PBS and fixed in 4% paraformaldehyde for 20 min, permeabilized with 0.2% Triton X-100 in PBS for 30 min at room temperature, and then blocked with goat serum at room temperature for 20 min. After blocking, tissue specimens or cell samples were incubated with primary antibodies overnight at 4°C. Then, the tissue specimens or cell samples were incubated with secondary antibodies in the dark for 1 h at room temperature; then, the nuclei was counterstained with 4'-6-diamidino-2-phenylindole (DAPI, Invitrogen, USA). Images were captured using a confocal microscope (Olympus, Japan).

Luciferase reporter assay

HEK-293T cells were seeded in 24-well plates. Then, the cells were cotransfected with either control mimics, miR-22 mimics or psiCHECK™-ERα 3'UTR using Lipofectamine 3000. Thirty-six hours later, the cells were lysed in passive lysis buffer (Promega, USA), and the renilla luciferase activity and firefly luciferase activity were measured respectively. Each group was analyzed in triplicate.

Co-culture assay

The co-culture assay was established using transwell membranes (pores 0.4 µm, Merck Millipore, USA) in a 24-well format. CAFs were on the up-chamber and then breast cancer cells below the membranes were ready for further cytological experiments after co-culture.

Cell proliferation assay

The viability of cells was measured by Cell Counting Kit-8 (CCK-8) solution (7sea biotech, China). Briefly, breast cancer cells were seeded on 24-well plates (Corning, USA) and incubated at 37°C overnight. The CCK-8 reagents were then added to a subset of wells. After the cells were incubated for 2 h at 37°C, we quantified the absorbance at 450 nm using a microplate reader (Bio-Rad, USA). Each group was made in triplicate. The cell viability (%) was calculated by the following formula: Cell viability (%) = (OD drug - OD blank)/(OD control - OD blank) × 100%.

Colony formation assay

The drug sensitivity to tamoxifen was also measured by colony formation assay. Breast cancer cells were plated in 6-well plates at a concentration of $1*10^3$ cell /well. The medium with 4-hydroxytamoxifen was replaced every 5 days. After two weeks cells were fixed by methanol and stained with crystal violet.

Biotin miRNA pull-down assay

Biotinylated single-stranded miR-22, mutated miR-22 and poly(G) were synthesized by Sangon Biotech (Shanghai, China). Briefly, lysates from $CD63^+$ CAFs or their exosomes were incubated overnight at 4 °C with wild-type or mutated miR-22 containing a biotin modification attached to the 5′ end. Washed streptavidin agarose beads (Invitrogen, USA) were added to each binding reaction, which was further

incubated at 4 °C for 4 h. The precipitates were washed three times, boiled in SDS buffer, and subjected to Western blotting. The sequence of the biotinylated poly(G) is (5′-GGGGGGGGGGGGGGGGGGGG-3′).

RIP assay

RIP assays were performed using an EZ-Magna RIP RNA-Binding Protein Immunoprecipitation kit (Millipore, USA). Briefly, $CD63^+$ CAFs were collected and lysed in ice-cold lysis buffer supplemented with protease inhibitors, RNase inhibitors, and PMSF. Lysates were centrifuged at 14,000×g for 15 min, and part of the lysate was saved as input. The protein extract was incubated with anti-SFRS1 antibody or negative control IgG overnight at 4 °C. Approximately 25 μl of Protein A/G Agarose beads was then added and incubated with the extracts at 4 °C for 4 h. Then, the beads were washed three times before the amounts of coimmunoprecipitated miRNAs were quantified by real-time PCR and normalized against the input from the same cells. Each group was assessed in triplicate.

Transcription factor activation profiling plate array

The activation of multiple TFs in $CD63^+$ CAFs and $CD63^+$ -depleted CAFs was screened using a TF Activation Profiling Plate Array (Signosis Inc, USA). In brief, the nuclear extracts of cells were preincubated with a biotin-labeled TF probe mix at room temperature for 30 min to form TF/DNA complexes. Free probes were filtered using an isolation column provided by the manufacturer. The eluted TF/DNA

complexes were denatured at 98 °C for 5 min and then submitted for hybridization analysis on the provided 96-well plate, which was specifically precoated with the complementary sequences of all the probes. Next, the well contents were hybridized in the plate at 42 °C overnight. Captured DNA probes were then further developed with a streptavidin-HRP conjugate. Luminescence was reported as relative light units (RLUs) on a TECAN M200 microplate luminometer (TECAN, Switzerland).

Gene set enrichment analysis (GSEA)

Normalized expression data were analyzed and visualized with GSEA software (version 2.2.0, http://www.broadinstitute.org/gsea). Normalized enrichment scores (NESs), *P* value and *q* value were calculated for comparison. Interrogated signatures include HALLMARK gene sets from the MSigDB database v5.1 release. "SMID_BREAST_CANCER_LUMINAL_A_UP" (M7517, with 85 genes) is genes up-regulated in the luminal A subtype of breast cancer; "SMID_BREAST_CANCER_LUMINAL_A_DN" (M13072, with 18 genes) is genes down-regulated in the luminal A subtype of breast cancer; "FRASOR_RESPONSE_TO_SERM_OR_FULVESTRANT_DN" (M11250, with 49 genes) is genes down-regulated in MCF-7 cells (breast cancer) by selective estrogen receptor modulators (SERM) 4-hydroxytamoxifen, raloxifene, or ICI 182780; "FRASOR_RESPONSE_TO_SERM_OR_FULVESTRANT_UP" (M8108, with 23 genes) is genes up-regulated in MCF-7 cells (breast cancer) by selective estrogen receptor modulators (SERM) 4-hydroxytamoxifen, raloxifene, or ICI 182780.

Kaplan–Meier plotter analysis

Survival analysis based on miR-22 expression in ERα positive breast cancer was performed on the Kaplan–Meier plotter website (www.kmplot.com), an online database that can assess the effect of 54,675 genes on the prognosis of breast cancer, ovarian cancer, lung cancer and gastric cancer patients (from TCGA or METABRIC).

Overall survival analysis of the CD63$^+$ CAF gene signature

Overall survival analysis based on CD63$^+$ CAF gene signature expression in breast cancer was performed with the Gene Expression Profiling Interactive Analysis browser (gepia.cancer-pku.cn/index.html). It is online database, where we can perform survival analysis based on the expression status of a multi-gene signature. Here the combined 5 genes (CD63, Col1a1, Col3a1, Thy1 and FAP) were used. The case with high CD63$^+$ CAF gene signature expression was associated with high abundance of CD63$^+$ CAFs in the tumor tissue.

GEO Datasets

The published breast cancer expression data set was extracted from Gene Expression Omnibus (GEO). GSE75688 is a published breast cancer expression data of Single cell RNA sequencing of human primary breast cancer. All single-cell mRNA expression profiles were acquired from 11 patients with distinct molecular subtypes (BC01-BC02, estrogen receptor positive (ER+); BC03, double positive (ER+ and

HER2+); BC04-BC06, human epidermal growth factor receptor 2 positive (HER2+); BC07-BC11, triple-negative breast cancer (TNBC)).

GSE83292 is a published breast cancer expression data of differential miRNA expression profiles of primary and relapse lesions of breast cancer patients receiving tamoxifen. In this dataset, 6 primary tumor samples and 6 matched recurrent/metastatic lesion samples from six ER+ breast cancer patients who relapsed after tamoxifen treatment were analyzed by microRNA array.

CAFs isolation

CAFs were isolated from breast cancer tissues obtained from surgery. Briefly, tissues were digested by Tumor Dissociation Kit (Miltenyi Biotec) according to the manufacturer's instructions. In order to isolate CAFs, the single-cell suspension was incubated without shaking for 5 min at room temperature, followed by the separation of stromal cell-enriched supernatant to a new tube. The stromal fraction was collected by centrifuge at 250×g for 5 min. Then magnetic-activated cell sorting (MASC) with anti-FSP (fibroblast specific protein) to purify the primary fibroblast. The CAFs were then cultured in RPMI-1640 with 10% FBS. The first to fifth passages of CAFs were used in our experiments.

Isolation of $CD63^+$ CAFs

Magnetic-activated cell sorting (MASC) with anti-CD63 is used to isolate $CD63^+$ CAFs. Briefly, total CAFs were digested into single cell suspension and washed twice

with PBS. Then the cells were resuspended and incubated with anti-CD63 MicroBeads at 4°C for 30 min. Then, cells were washed by PBS. The labeled cells were loaded onto LS Column, which was placed in the magnetic field. The magnetically labeled $CD63^+$ CAFs were retained within the column, while the unlabeled run through the column.
